# Synthesis of xanthohumol and xanthohumol-d_3_ from naringenin†

**DOI:** 10.1039/d1ra05443k

**Published:** 2021-08-31

**Authors:** Joanna Andrusiak, Kinga Mylkie, Małgorzata Wysocka, Jacek Ścianowski, Andrzej Wolan, Marcin Budny

**Affiliations:** Department of Organic Chemistry, Faculty of Chemistry, Nicolaus Copernicus University Gagarina 7 87-100 Toruń Poland; Synthex Technologies Sp. z o.o. Gagarina 7/134B 87-100 Toruń Poland budny@synthex.com.pl; Department of Biomedical and Polymer Chemistry, Faculty of Chemistry, Nicolaus Copernicus University Gagarina 7 87-100 Toruń Poland; Noctiluca S.A. Gagarina 7/41B 87-100 Toruń Poland

## Abstract

A six-step synthesis of xanthohumol (1a) and its d_3_-derivative (1b) from easily accessible naringenin is reported. The prenyl side chain was introduced by Mitsunobu reaction followed by the europium-catalyzed Claisen rearrangement and base-mediated opening of chromanone gave access to an α,β-conjugated ketone system. Compound 1b was used as an internal standard in stable isotope dilution assays of 1a in two Polish beers.

## Abstract 2

A six-step synthesis of xanthohumol and its d_3_-derivative from easily accessible naringenin is reported.

## Graphical abstract

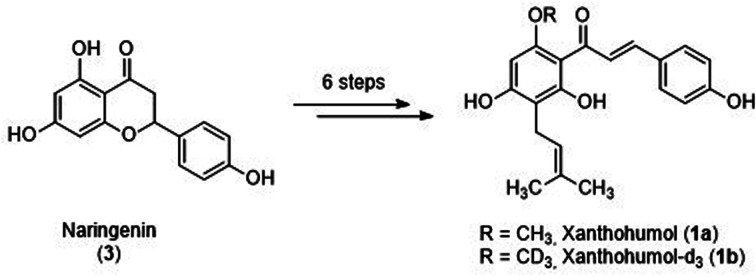

Xanthohumol (1a, Scheme 1A) is a naturally occurring prenylated chalcone produced by lupulin glands in female inflorescences of hop plants.^1^ In recent years, 1a has attracted significant attention due to its vast range of biological activities including cancer-preventive, antioxidative, anti-inflammatory, and antiviral.^2–8^ Such properties combined with low toxicity to the human body make 1a a prospective therapeutic agent, diet supplement, or ingredient of cosmetics.^9^ Although 1a was isolated from natural sources in 1913,^10^ the first synthesis of this compound was reported as late as in 2007.^11^ Several other syntheses have been reported since then, but only minor improvements have been achieved.^12–14^

**Scheme 1:**
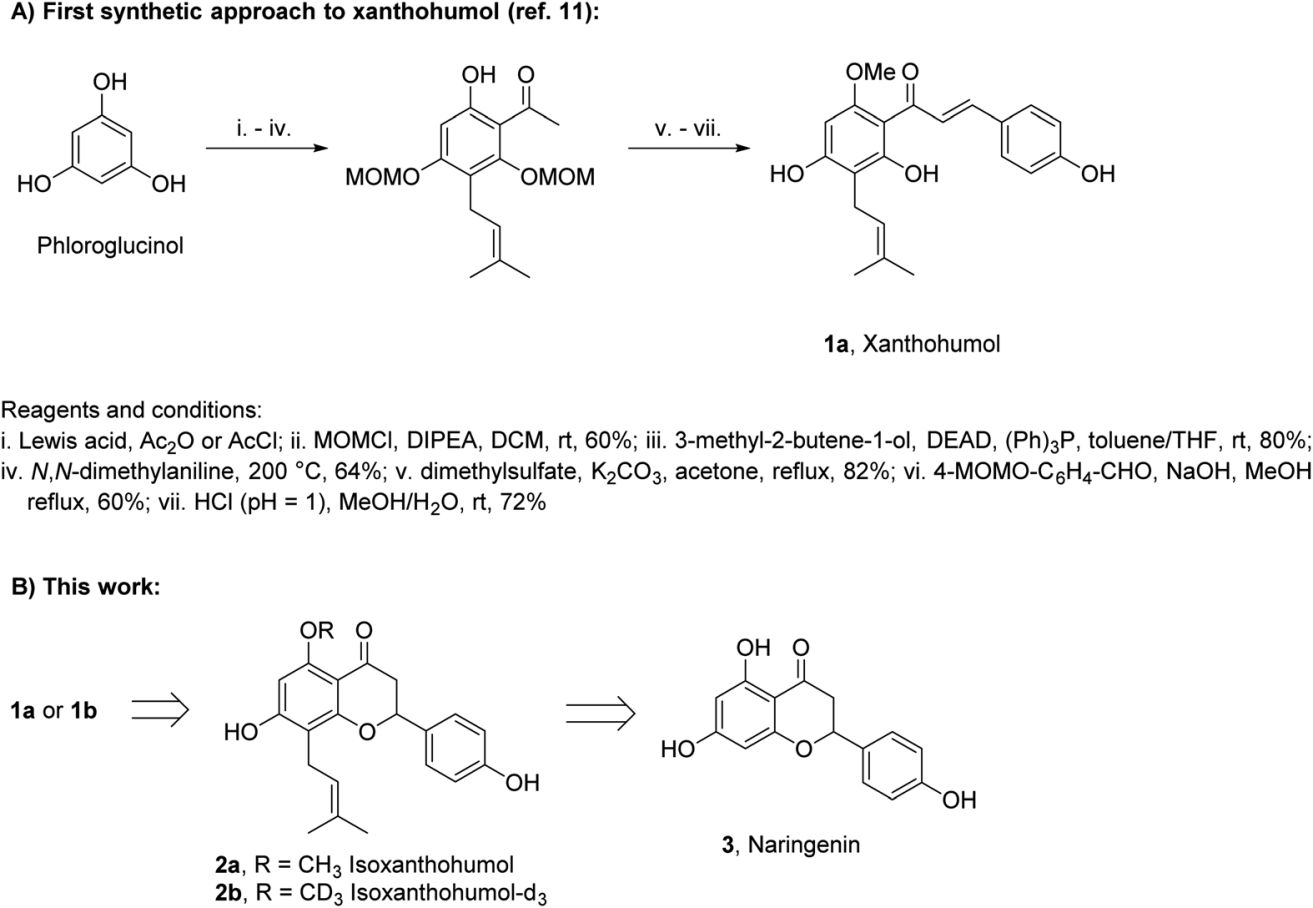
The background of the study.

Bioactive compounds labeled with stable isotopes (deuterium, carbon-13) are widely applied in metabolomic studies for tracking metabolic pathways and as internal standards in stable isotope dilution assays.^15,16^ Deuterated compounds are also considered as attractive drug candidates due to the influence of the kinetic isotope effect on pharmacokinetics.^17–19^ Although approaches to ^13^C-enriched xanthohumol^20,21^ and hydrogen/deuterium exchange in 1a^22^ were reported, no scalable and cost-effective synthesis of the deuterium-labeled derivative of 1a (*i.e.*1b) has been disclosed to date.

Two main challenges have to be faced in the synthesis of 1a: (i) construction of a pentasubstituted aromatic ring containing a prenyl side chain and (ii) selection of suitable protecting groups for phenols. In the case of (i), phloroglucinol is used as a precursor and an acyl-substituent is introduced by Friedel–Crafts acylation with a subsequent Claisen–Schmidt condensation. The prenyl side-chain is introduced by Mitsunobu alkylation, followed by Claisen rearrangement. In the case of (ii), acid-sensitive alkoxymethyl protecting groups, removable under conditions in which 1a does not cyclize to isoxanthohumol (2a), are used most often (Scheme 1A).

In this study, we have developed a synthetic approach for the formation of 1a and its deuterated analog 1b. We envisioned that both 1a and 1b can be directly obtained by the base-promoted chromanone ring-opening of 2a or 2b, which in turn can be obtained from easily accessible naringenin (3) (*ca.* 1 $/1 g) *via* two-step prenylation and Williamson etherification of the phenolic OH (Scheme 1B). The use of 3 as the starting material is beneficial as only one prenyl substituent has to be introduced.

The synthetic route leading to 1a and 1b is depicted in Scheme 2. Our synthesis commenced from naringenin (3), which was selectively converted to diester 4 (Ac_2_O, pyridine). *O*-Alkylation of 4 under Mitsunobu conditions (3-methyl-2-butene-1-ol, Ph_3_P, DIAD), followed by the catalytic Claisen rearrangement of 5 (Eu(fod)_3_, 1,2-dichloroethane, 80 °C) afforded prenyl-derivative 6. Notably, performing the latter reaction in 1,2-dichloroethane above its boiling point was superior in comparison to earlier reports.^23–26^ Alkylation of 6 (CH_3_I, Ag_2_O or CD_3_I, Ag_2_O) afforded 7a/7b in good yields. An alternative approach to 7b involving the alkylation of phenolic OH under Mitsunobu conditions (CD_3_OD, Ph_3_P, DIAD) required a large excess of reagents and afforded the product in moderate yield. Basic hydrolysis (KOH, MeOH) of esters afforded isoxanthohumols 2a/2b. Although the chromane ring was stable during the hydrolysis, it could be opened under more harsh conditions (DBU, DMF, 70 °C),^27,28^ leading to 1a/1b in good yields after a mild acidic workup.

**Scheme 2:**
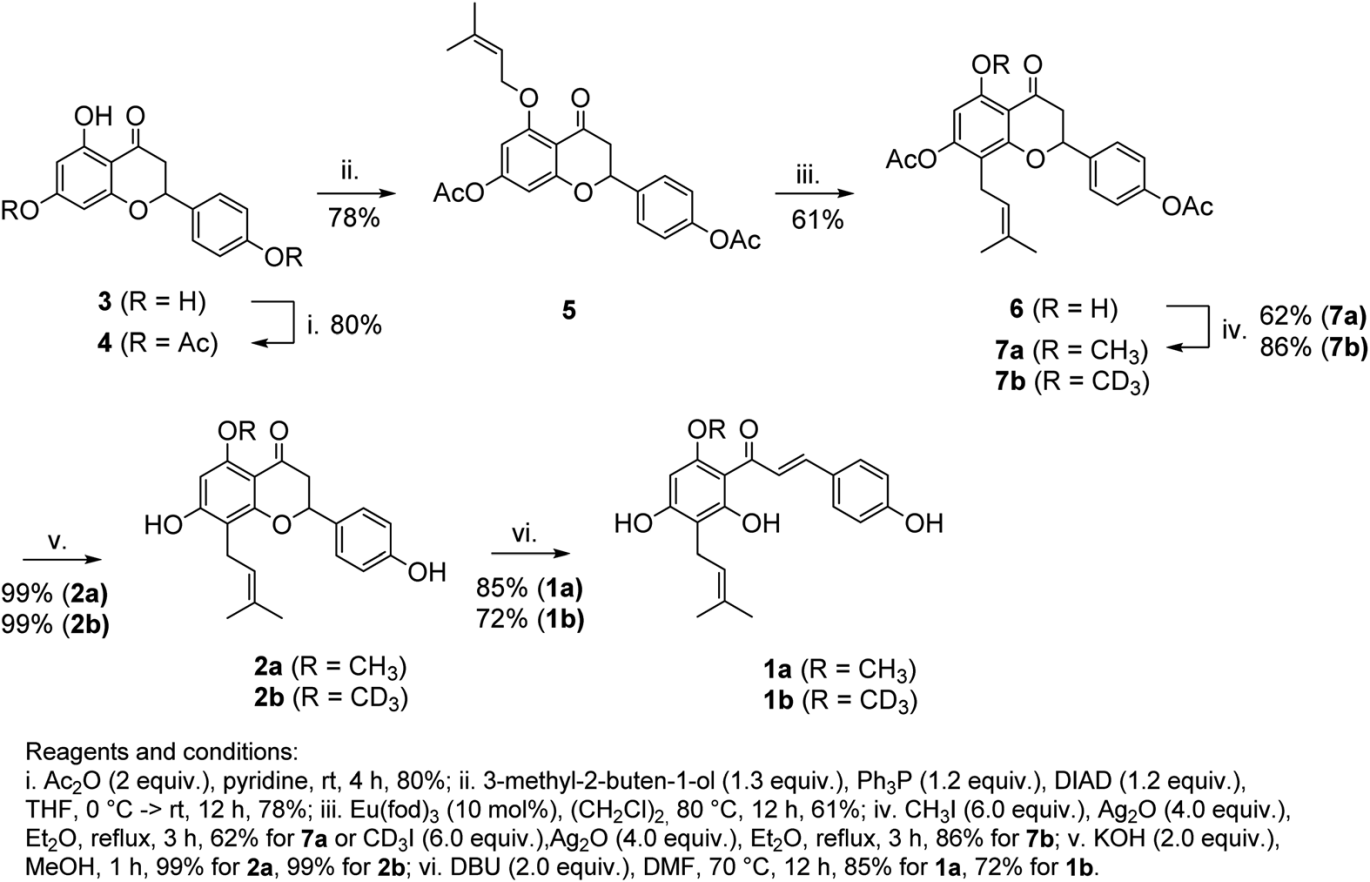
The synthetic route to 1a and 1b.

With 1a and 1b in hand, we investigated their MS-fragmentation patterns in electrospray ionization in positive and negative ion modes. The MRM transitions were found by an automatic procedure and they are listed in Table 1 (see ESI† for details). In positive ion mode, the most intensive product ions in fragmentation of 1a were ions with *m*/*z* values of 178.9, 299.0, 113.0, and 150.9. Corresponding ions with *m*/*z* +3 values can be found in the fragmentation of 1b. On the other hand, in the negative ion mode, the same product ions are observed both in the case of 1a and 1b, indicating that the CH_3_/CD_3_ groups were lost during fragmentations.

**Table 1:** The MRM transitions of 1a and 1b

Ionization mode	1a		1b	
	Precursor ion	Product ion	Precursor ion	Product ion
ESI(+)	355.0	178.9	358.0	182.0
		299.0		302.0
		113.0		115.9
		150.9		107.9
		93.0		154.0
ESI(−)	353.0	119.1	356.0	119.1
		233.0		236.0
		295.1		295.2
		218.2		175.0
		175.0		218.1
		189.2		168.2

One of the criteria for an effective internal standard is the coelution of the labeled and non-labeled compounds during the HPLC analysis. This is particularly important in case of deuterium-labeled compounds as with the increase in the number of deuterons in the molecule, retention times may be extended. The retention times of 1a and 1b under different HPLC conditions are listed in Table 2. Notably, coelution of 1a and 1b is observed in case of the XB-C18 stationary phase (entry 1). Minor differences were observed when separation was attempted on C18-PFP (entry 2) and polar-C18 (entry 3) stationary phases.

**Table 2:** Comparison of the retention times of 1a and 1b under different HPLC conditions

Entry	Conditions	Retention time [min]	
		1a	1b
1	Column: XB-C18, 100 × 3.0 mm, 2.6 μm, 100 Å; flow: 0.55 mL min^−1^; oven: 35 °C; gradient MeOH/0.1% HCO_2_H_(aq)_: from 5% MeOH to 95% MeOH	20.030	20.034
2	Column: Ace 5 C18-PFP, 250 × 4.6 mm; flow: 1.0 mL min^−1^, oven: 35 °C; isocrat. MeOH/0.1% HCO_2_H_(aq)_: 80 : 20	19.615	19.700
3	Column: polar-C18, 100 × 3.0 mm, 2.6 μm, 100 Å; flow: 0.55 mL min^−1^; oven: 35 °C; isocrat.: MeOH/0.1% HCO_2_H_(aq)_: 65 : 35	6.590	6.520

As an example of applications, compound 1b was used as an internal standard in a stable isotope dilution assay of xanthohumol (1a) in two Polish beers (determined concentrations: 0.4069 mg L^−1^ and 0.5488 mg L^−1^, respectively). The developed MRM method allowed for the direct analysis of 1a and any preconcentration of the analyte was not needed.

In conclusion, we have developed a six-step synthesis of xanthohumol (1a) and its deuterated analog 1b from naringenin (3) in total 19.8% yield for 1a and 23.3% for 1b. In a key step, isoxanthohumols 2a/2b were converted to the target compounds under basic conditions. The overall synthetic route was scalable and was used in the synthesis of 1a on a 5 g scale. The MRM transitions of 1b and its coelution with 1a makes 1b a suitable internal standard for the stable isotope dilution assay.

## Experimental section

Tetrahydrofuran (THF) and *N*,*N*-dimethylformamide (DMF) were dried over activated molecular sieves 4 Å. Other solvents and reagents were of analytical grade and were used as received without further purification. NMR spectra were recorded on Bruker Avance III 300 MHz, 400 MHz, and 700 MHz spectrometers. Chemical shifts are reported as *δ* values in parts per million relative to the residual solvent signal (CDCl_3_: *δ* = 7.24 ppm for ^1^H and 77.23 ppm for ^13^C; CD_3_OD: *δ* = 3.31 ppm for ^1^H and 49.15 ppm for ^13^C). Coupling constants are in hertz (Hz). The following abbreviations are used for spin multiplicity: s = singlet, d = doublet, t = triplet, q = quartet, m = multiplet, and br = broad. HPLC-ESI MS analyses were performed on a triple quadrupole Shimadzu LCMS 8030. Infrared spectra were recorded on a PerkinElmer UATR two instrument and are reported in cm^−1^. Melting points were determined in open glass capillaries and are uncorrected. Silica Gel 60, Merck 230–400, was used for preparative column chromatography. Sigma-Aldrich TLC plates (silica gel on Al foil with fluorescent indicator 254 nm) were used for analytical TLC. UV lamp (*λ* = 254 nm) and solution of phosphomolybdic acid in ethanol were used for the visualization of TLC plates.

### 4-(7-Acetoxy-5-hydroxy-4-oxochroman-2-yl)phenyl acetate (4)

To a suspension of naringenin (3) (1.0 equiv., 73.5 mmol, 20.0 g) in pyridine (60 mL), acetic anhydride (2.0 equiv., 147.0 mmol, 15.0 g) was added portionwise. The resulting solution was stirred until the complete consumption of the starting material. The reaction was quenched with conc. HCl (60 mL) and water (200 mL), extracted with EtOAc (3 × 100 mL), and washed with water (3 × 100 mL) and brine (100 mL). The extract was dried over anhydrous MgSO_4_, filtered, and concentrated under reduced pressure. The residue was recrystallized from MeOH to afford 4 (20.94 g, 80%) as a white solid.

Mp. 143–144 °C; IR (neat, cm^−1^): 2964, 1742, 1650, 1370, 1276, 1210, 1076, 1014, 840, 769; ^1^H NMR (700 MHz, CDCl_3_), *δ* (ppm): 11.81 (s, 1H), 7.46–7.43 (m, 2H), 7.16–7.13 (m, 2H), 6.30 (d, *J* = 2.1 Hz, 1H), 6.29 (d, *J* = 2.1 Hz, 1H), 5.44 (dd, *J*_1_ = 13.4 Hz, *J*_2_ = 2.9 Hz, 1H), 3.08 (dd, *J*_1_ = 17.2 Hz, *J*_2_ = 13.4 Hz, 1H), 2.85 (dd, *J*_1_ = 17.2 Hz, *J*_2_ = 2.9 Hz, 1H), 2.30 (s, 3H), 2.27 (s, 3H). ^13^C NMR (75 MHz, CDCl_3_), *δ* (ppm): 196.9, 169.4, 168.4, 163.6, 162.4, 158.7, 151.3, 135.8, 127.5, 122.3, 106.4, 103.6, 101.9, 79.0, 43.8, 21.4, 21.3; anal. calcd for C_19_H_16_O_7_: C, 64.04; H, 4.53. Found: C, 64.17; H, 4.60.

### 4-(7-Acetoxy-5-((3-methylbut-2-en-1-yl)oxy)-4-oxochroman-2-yl)phenyl acetate (5)

To a solution of 4 (1 equiv., 30.3 mmol, 10.792 g) in anhydrous THF (270 mL), 3-methylbut-2-en-1-ol (1.3 equiv., 39.4 mmol, 3.39 g) and triphenylphosphine (1.2 equiv., 36.4 mmol, 9.55 g) were added. The resulting solution was cooled to 0 °C, and then DIAD (1.2 equiv., 36.4 mmol, 7.36 g) was added portionwise. The resulting mixture was stirred at 0 °C for 30 min and then at room temperature (rt) overnight. The reaction mixture was concentrated under reduced pressure. The residue was dissolved in MTBE (250 mL), and the resulting solution was stirred at 0 °C for 30 min. The precipitated triphenylphosphine oxide was filtered off and the solution was concentrated under a reduced pressure. The residue was purified by flash chromatography (petroleum ether-ethyl acetate = 70 : 30 → 60 : 40) affording 5 (10.03 g, 78%) as a pale yellow solid.

Mp. 113–116 °C; IR (neat, cm^−1^): 2979, 1764, 1680, 1596, 1373, 1197, 1108, 1076, 1031, 904, 843; ^1^H NMR (700 MHz, CDCl_3_), *δ* (ppm): 7.46–7.43 (m, 2H), 7.13–7.11 (m, 2H), 6.40 (d, *J* = 2.1 Hz, 1H), 6.29 (d, *J* = 2.1 Hz, 1H), 5.53–5.49 (m, 1H), 5.41 (dd, *J*_1_ = 13.2 Hz, *J*_2_ = 2.7 Hz, 1H), 4.62–4.56 (m, 2H), 2.99 (dd, *J*_1_ = 16.4 Hz, *J*_2_ = 13.2 Hz, 1H), 2.80 (dd, *J*_1_ = 16.4 Hz, *J*_2_ = 2.7 Hz, 1H), 2.29 (s, 3H), 2.28 (s, 3H), 1.77 (br s, 3H), 1.72 (br s, 3H). ^13^C NMR (175 MHz, CDCl_3_), *δ* (ppm): 189.2, 169.6, 168.6, 163.9, 161.4, 156.6, 151.0, 138.5, 136.3, 127.5, 122.2, 119.1, 109.8, 103.4, 100.1, 78.9, 66.3, 46.0, 26.0, 21.4, 21.3, 18.6; anal. calcd for C_24_H_24_O_7_: C, 67.91; H, 5.70. Found: C, 68.01; H, 5.68.

### 4-(7-Acetoxy-5-hydroxy-8-(3-methylbut-2-en-1-yl)-4-oxochroman-2-yl)phenyl acetate (6)

In a 50 mL pressure vial, 5 (1 equiv., 6.96 mmol, 2.95 g), Eu(fod)_3_ (0.1 equiv., 0.696 mmol, 722 mg) and 1,2-dichloroethane (6 mL) were placed. The resulting mixture was stirred at 80 °C (temp. of the oil bath) overnight. Then, the mixture was cooled to rt and directly purified by flash chromatography (petroleum ether-ethyl acetate = 90 : 10 → 70 : 30) to afford 6 (1.80 g, 61%) as a white solid.

Mp. 143–144 °C; IR (neat, cm^−1^): 2975, 1763, 1637, 1428, 1372, 1189, 1066, 1012, 896; ^1^H NMR (700 MHz, CDCl_3_), *δ* (ppm): 11.70 (s, 1H), 7.46–7.43 (m, 2H), 7.16–7.13 (m, 2H), 6.29 (s, 1H), 5.43 (dd, *J*_1_ = 13.3 Hz, *J*_2_ = 2.9 Hz, 1H), 5.06–5.01 (m, 1H), 3.18–3.10 (m, 2H), 3.06 (dd, *J*_1_ = 17.1 Hz, *J*_2_ = 13.3 Hz, 1H), 2.87 (dd, *J*_1_ = 17.1 Hz, *J*_2_ = 2.9 Hz, 1H), 2.30 (s, 3H), 2.28 (s, 3H), 1.64–1.62 (m, 3H), 1.58–1.56 (m, 3H). ^13^C NMR (175 MHz, CDCl_3_), *δ* (ppm): 197.3, 169.4, 168.4, 161.1, 160.0, 157.0, 151.2, 136.1, 132.1, 127.4, 122.2, 121.8, 113.8, 106.8, 104.3, 78.9, 43.8, 25.8, 22.9, 21.3, 21.1, 18.0; anal. calcd for C_24_H_24_O_7_: C, 67.91; H, 5.70; N, 14.42. Found: C, 67.94; H, 5.82.

### 4-(7-Acetoxy-5-methoxy-8-(3-methylbut-2-en-1-yl)-4-oxochroman-2-yl)phenyl acetate (7a)

To a suspension of 6 (1 equiv., 9.46 mmol, 4.012 g) and freshly prepared Ag_2_O (4 equiv., 37.85 mmol, 8.770 g) in Et_2_O (95 mL), MeI (6 equiv., 56.772 mmol, 8.058 g, 3.66 mL) was added. The resulting mixture was refluxed for 3 h. Then, the reaction mixture was cooled to rt, silver salts were filtered off, and the filtrate was concentrated under reduced pressure. The residue was purified by flash chromatography (petroleum ether-ethyl acetate = 60 : 40) affording 7a (2.59 g, 62%) as a white solid.

Mp. 140–141 °C; IR (neat, cm^−1^): 2962, 1753, 1649, 1592, 1369, 1203, 1098, 1055, 902, 834; ^1^H NMR (700 MHz, CDCl_3_), *δ* (ppm): 7.46–7.42 (m, 2H), 7.14–7.10 (m, 2H), 6.28 (s, 1H), 5.41 (dd, *J*_1_ = 13.3 Hz, *J*_2_ = 2.8 Hz, 1H), 5.07–5.02 (m, 1H), 3.86 (s, 3H), 3.21–3.15 (m, 2H), 2.98 (dd, *J*_1_ = 16.4 Hz, *J*_2_ = 13.3 Hz, 1H), 2.83 (dd, *J*_1_ = 16.4 Hz, *J*_2_ = 2.8 Hz, 1H), 2.31 (s, 3H), 2.30 (s, 3H), 1.64–1.63 (m, 3H), 1.58–1.56 (m, 3H). ^13^C NMR (175 MHz, CDCl_3_), *δ* (ppm): 190.1, 169.6, 168.9, 161.9, 159.6, 154.8, 150.9, 136.5, 132.2, 127.4, 122.1, 121.7, 115.4, 109.9, 99.6, 78.8, 56.5, 45.8, 25.9, 23.2, 21.4, 21.1, 18.00; anal. calcd for C_25_H_26_O_7_: C, 68.48; H, 5.98. Found: C, 68.21; H, 6.11.

### 4-(7-Acetoxy-5-(methoxy-d_3_)-8-(3-methylbut-2-en-1-yl)-4-oxochroman-2-yl)phenyl acetate (7b)

The same procedure as for compound 7a was used. Starting from 6 (2.36 mmol, 1.0 g) 7b (0.89 g, 86%) as a pale yellow solid was obtained.

Mp. 138–139 °C, IR (neat, cm^−1^): 1754, 1685, 1593, 1362, 1204, 1091, 902. ^1^H NMR (700 MHz, CDCl_3_), *δ* (ppm): 7.46–7.42 (m, 2H), 7.14–7.10 (m, 2H), 6.27 (s, 1H), 5.41 (dd, *J*_1_ = 13.1 Hz, *J*_2_ = 2.8 Hz, 1H), 5.07–5.03 (m, 1H), 3.21–3.14 (m, 2H), 2.98 (dd, *J*_1_ = 16.5 Hz, *J*_2_ = 13.2 Hz, 1H), 2.83 (dd, *J*_1_ = 16.5 Hz, *J*_2_ = 2.8 Hz, 1H), 2.30 (s, 3H), 2.29 (s, 3H), 1.64–1.62 (m, 3H), 1.58–1.56 (m, 3H). ^13^C NMR (175 MHz, CDCl_3_), *δ* (ppm): 189.9, 169.4, 168.8, 161.8, 159.7, 154.9, 151.0, 136.6, 132.2, 127.3, 122.1, 121.8, 115.4, 110.0, 99.6, 78.8, 78.8, 45.8, 25.8, 23.2, 21.3, 21.1, 18.0; anal. calcd for C_25_H_23_D_3_O_7_: C, 68.01; H, 6.62. Found: C, 68.07; H, 6.60.

### 7-Hydroxy-2-(4-hydroxyphenyl)-5-methoxy-8-(3-methylbut-2-en-1-yl)chroman-4-one (2a)

To a solution of 7a (1 equiv., 4.349 mmol, 1.905 g) in MeOH (30 mL), KOH (2 equiv., 8.699 mmol, 488 mg) was added. The reaction mixture was stirred until the complete consumption of the starting material. The reaction mixture was neutralized with 2 M HCl, extracted with EtOAc (3 × 60 mL), washed with water (2 × 30 mL) and brine (30 mL), dried over anhydrous MgSO_4_, filtered and concentrated under reduced pressure. The residue was purified by flash chromatography (petroleum ether-ethyl acetate = 50 : 50) to afford 2a (1.524 g, 99%) as a pale yellow solid.

Mp. 157–161 °C; IR (neat, cm^−1^): 3150, 1646, 1590, 1452, 1410, 1349, 1270, 1088, 827; ^1^H NMR (700 MHz, CD_3_OD), *δ* (ppm): 7.33–7.28 (m, 2H), 6.83–6.79 (m, 2H), 6.11 (s, 1H), 5.28 (dd, *J*_1_ = 12.8 Hz, *J*_2_ = 3.0 Hz, 1H), 5.15–5.12 (m, 1H), 3.79 (s, 3H), 3.24–3.17 (m, 2H), 2.97 (dd, *J*_1_ = 16.7 Hz, *J*_2_ = 12.8 Hz, 1H), 2.66 (dd, *J*_1_ = 16.7 Hz, *J*_2_ = 3.0 Hz, 1H), 1.62 (s, 3H), 1.56 (s, 3H). ^13^C NMR (700 MHz, CD_3_OD), *δ* (ppm): 193.0, 164.4, 164.0, 159.0, 131.8, 131.7, 129.0, 124.1, 116.4, 110.2, 106.1, 93.8, 80.2, 56.1, 46.4, 26.0, 22.9, 18.0; anal. calcd for C_21_H_22_O_5_: C, 71.17; H, 6.26. Found: C, 71.38; H, 6.42.

### 7-Hydroxy-2-(4-hydroxyphenyl)-5-(methoxy-d_3_)-8-(3-methylbut-2-en-1-yl)chroman-4-one (2b)

The same procedure as for compound 2a was used. Starting from 7b (4.33 mmol, 1.799 g) 2b (1.530 g, 99%) as a pale yellow solid was obtained.

Mp. 150–151 °C; IR (neat, cm^−1^): 3164, 1591, 1509, 1422, 1359, 1259, 1095, 823; ^1^H NMR (700 MHz, CD_3_OD), *δ* (ppm): 7.33–7.28 (m, 2H), 6.83–6.78 (m, 2H), 6.10 (s, 1H), 5.27 (dd, *J*_1_ = 12.8 Hz, *J*_2_ = 2.9 Hz, 1H), 5.18–5.11 (m, 1H), 3.24–3.17 (m, 2H), 2.97 (dd, *J*_1_ = 16.5 Hz, *J*_2_ = 12.8 Hz, 1H), 2.65 (dd, *J*_1_ = 16.5 Hz, *J*_2_ = 2.9 Hz, 1H), 1.61 (s, 3H), 1.56 (s, 3H). ^13^C NMR (100 MHz, CD_3_OD), *δ* (ppm): 193.0, 164.3, 164.0, 162.0, 158.9, 131.8, 131.7, 129.0, 124.0, 116.4, 110.1, 106.1, 93.6, 80.1, 46.4, 26.1, 22.9, 18.0; anal. calcd for C_21_H_19_D_3_O_5_: C, 70.57; H, 7.05. Found: C, 70.46; H, 6.96.

### (*E*)-1-(2,4-Dihydroxy-6-methoxy-3-(3-methylbut-2-en-1-yl)phenyl)-3-(4-hydroxyphenyl)prop-2-en-1-one (1a)

To a solution of 2a (1 equiv., 4.349 mmol, 1.544 g) in DMF (20 mL), DBU (2 equiv., 8.699 mmol, 1.324 g) was added dropwise and the reaction mixture was stirred at 70 °C for 12 h. Then, the reaction mixture was neutralized with 2 M HCl, extracted with EtOAc (4 × 60 mL), washed with water (3 × 30 mL) and brine (30 mL). The combined extracts were dried over anhydrous MgSO_4_, filtered and concentrated under reduced pressure. The residue was purified by flash chromatography (petroleum ether-ethyl acetate = 60 : 40) affording 1a (1.31 g, 85%) as orange solid.

Mp. 151–152 °C; IR (neat, cm^−1^): 3184, 2916, 1596, 1511, 1437, 1340, 1100, 824, 804; ^1^H NMR (700 MHz, CD_3_OD), *δ* (ppm): 7.80 (d, *J* = 15.6 Hz, 1H), 7.67 (d, *J* = 15.6 Hz, 1H), 7.51–7.49 (m, 2H), 6.85–6.81 (m, 2H), 6.02 (s, 1H), 5.22–5.18 (m, 1H), 3.09 (s, 3H), 3.23 (d, *J* = 7.1 Hz, 2H), 1.76 (s, 3H), 1.65 (s, 3H). ^13^C NMR (75 MHz, CD_3_OD), *δ* (ppm): 194.2, 166.3, 163.6, 161.2, 143.4, 131.5, 131.4, 128.6, 126.0, 124.4, 117.0, 109.6, 106.7, 91.8, 56.3, 26.1, 22.4, 18.0; anal. calcd for C_21_H_22_O_5_: C, 71.17; H, 6.26. Found: C, 71.03; H, 6.20.

### (*E*)-1-(2,4-Dihydroxy-6-(methoxy-d_3_)-3-(3-methylbut-2-en-1-yl)phenyl)-3-(4-hydroxyphenyl)prop-2-en-1-one (1b)

The same procedure as for compound 1b was used.

Starting from 2b (4.43 mmol, 1.581 g) 1b (1.141 g, 72%) as a pale yellow solid was obtained.

Mp. 149–151 °C, IR (neat, cm^−1^): 3302, 2915, 1521, 1439, 1337, 1213, 1157, 1098, 830; ^1^H NMR (700 MHz, CD_3_OD), *δ* (ppm): 7.79 (d, *J* = 15.5 Hz, 1H), 7.66 (d, *J* = 15.5 Hz, 1H), 7.51–7.46 (m, 2H), 6.85–6.79 (m, 2H), 6.01 (s, 1H), 5.22–5.18 (m, 1H), 3.23 (d, *J* = 7.0 Hz, 2H), 1.76 (s, 3H), 1.65 (s, 3H). ^13^C NMR (100 MHz, CD_3_OD), *δ* (ppm): 194.3, 166.3, 163.8, 162.6, 161.1, 143.4, 131.5, 131.4, 128.7, 126.1, 124.4, 117.0, 109.6, 106.7, 91.9, 26.1, 22.4, 18.0; anal. calcd for C_21_H_19_D_3_O_5_: C, 70.57; H, 7.05. Found: C, 70.55; H, 6.97.

## Conflicts of interest

The authors declare no conflict of interest.

## Supplementary Material

RA-011-D1RA05443K-s001
