# Supplementary material for: Synthesis of xanthohumol and xanthohumol-d3 from naringenin
Source: RSC Adv. 2021 Aug 31;11(46):28934–9. doi: 10.1039/d1ra05443k (PMC9038150; doi:10.1039/d1ra05443k)
Supplement: RA-011-D1RA05443K-s001 [file RA-011-D1RA05443K-s001.pdf]

Electronic Supplementary Material (ESI) for RSC Advances.
This journal is © The Royal Society of Chemistry 2021

Suplementary Data for

Synthesis of xanthohumol and xanthohumol-d$_3$ from naringenin

Joanna Andrusiak[a,b], Kinga Mylkie[c,d], Małgorzata Wysocka[b], Andrzej Wolan[a,b], Jacek Ścianowski[a], Marcin Budny[b]

[a] Department of Organic Chemistry, Faculty of Chemistry, Nicolaus Copernicus University, Gagarina 7, 87-100 Toruń, Poland; [b] Synthex Technologies Sp. z o.o., Gagarina 7/134B, 87-100 Toruń, Poland; [c] Department of Biomedical and Polymer Chemistry, Faculty of Chemistry, Nicolaus Copernicus University, Gagarina 7, 87-100 Toruń; [d] Noctiluca S.A., ul. Kościuszki 71/208; 87-100 Toruń, Polska

Contents

Suplementary Data for 1
The MRM transitions of **1a** and **1b** 2
Stable isotope dilution assays 2
Calibration curves 3
Typical MS chromatogram for calibration points 4
Typical MS chromatogram for beer sample 4
^{1}H and ^{13}C NMR spectra 4

The MRM transitions of **1a** and **1b**

Table S1. Details of the MRM transitions of **1a** and **1b**

Compound	Ionization mode	Precursor ion	Product ion	Q1 [V][a]	CE [eV][b]	Q3 [V][c]
**1a**	ESI(+)	**355.0**	**178.9**	**-17.0**	**-27.0**	**-29.0**
			**299.0**	**-17.0**	**-13.0**	**-29.0**
			113.0	-17.0	-48.0	-16.0
			150.9	-17.0	-44.0	-24.0
			93.0	-17.0	-55.0	-29.0
	ESI(-)	353.0	119.1	18.0	27.0	21.0
			233.0	18.0	19.0	23.0
			295.1	18.0	29.0	27.0
			218.2	18.0	25.0	21.0
			175.0	18.0	40.0	28.0
			189.2	18.0	29.0	17.0
**1b**	ESI(+)	**358.0**	**182.0**	**-26.0**	**-25.0**	**-30.0**
			**302.0**	**-26.0**	**-13.0**	**-29.0**
			115.9	-26.0	-49.0	-18.0
			107.9	-26.0	-55.0	-16.0
			154.0	-26.0	-45.0	-25.0
	ESI(-)	356.0	119.1	10.0	29.0	21.0
			236.0	10.0	19.0	23.0
			295.2	10.0	28.0	27.0
			175.0	10.0	39.0	30.0
			218.1	10.0	25.0	21.0
			168.2	10.0	28.0	30.0

[a] voltage on 1st quadrupole; [b] collision energy; [c] voltage on 3rd quadrupole; highlighted transitions were used for preparation of analytical HPLC-MS method

Stable isotope dilution assays

Stable isotope dilution assays were performed for two polish beers (Beer #1, Beer#2). A freshly opened beer was degassed for 30 min in ultrasonic cleaner. Then, to aliquots (1.0 mL) of degassed beer, samples of standard solution (**1b**, C = 5.720 mg/L) were added (0.1 mL, 0.2 mL, 0.3 mL, 0.4 mL, 0.5 mL). Prepared in such way samples together with beer samples were analyzed by HPLC-MS in MRM mode (Column: XB-C18, 100×3.0 mm, 2.6 µm, 100 Å; flow: 0.55 mL/min; oven: 35 °C; gradient MeOH/0.1% $HCO_2H_{(aq)}$: from 50% MeOH to 95% MeOH). Plots of peak areas versus standard concentrations in samples were sketched and concentration of **1a** was determined from the plot as absolute value of zero point.

Table S2. Stable isotope dilution assays

Analysis	Standard volume [mL]	Std. conc. in sample [mg/L]	Peak area
Beer #1		**Xanthohumol conc. [mg/L]**	**0.4069**
1	0.0	0	952552
2	0.1	0.520	2342036
3	0.2	0.953	3518620
4	0.3	1.320	4319572
5	0.4	1.634	4839326
6	0.5	1.907	5883749
Beer #2		**Xanthohumol conc. [mg/L]**	**0.5488**
1	0.0	0	1512201
2	0.1	0.520	3008853
3	0.2	0.953	4244305
4	0.3	1.320	5208824
5	0.4	1.634	6137508
6	0.5	1.907	6853290

Calibration curves

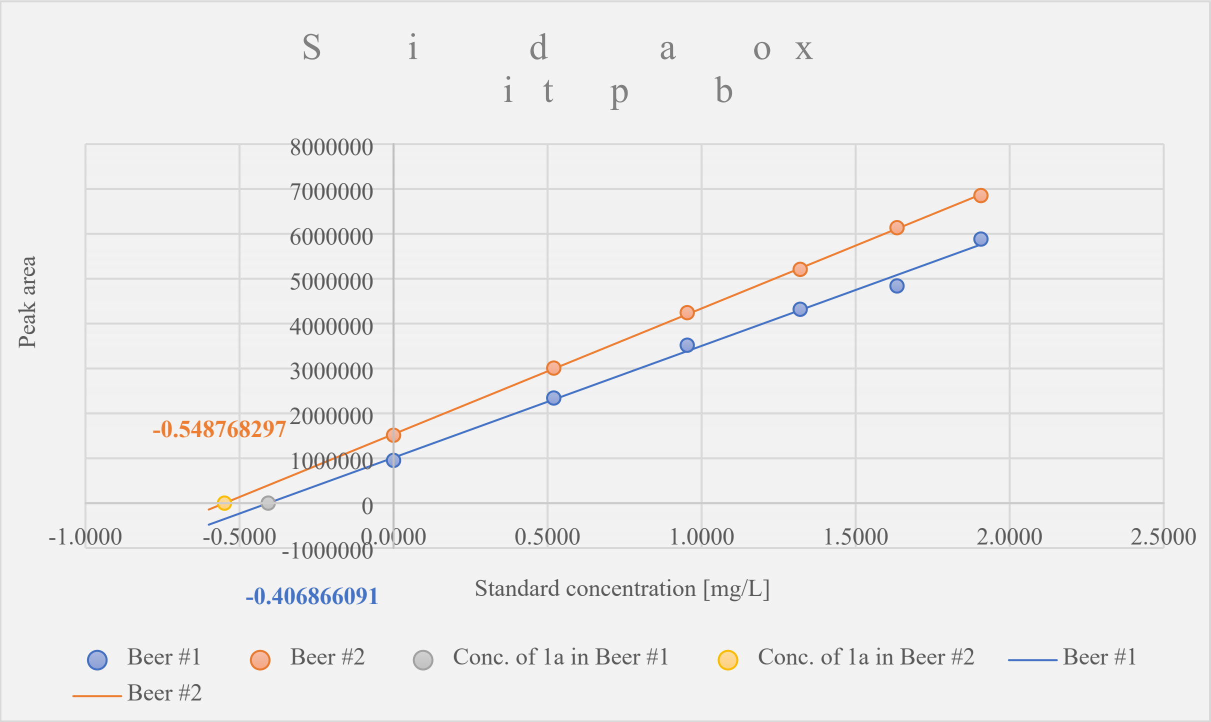

Typical MS chromatogram for calibration points

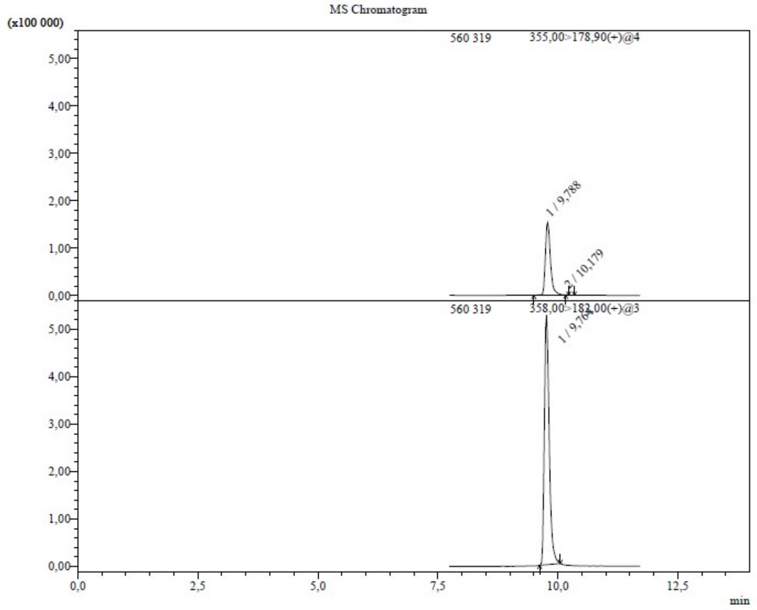

Typical MS chromatogram for beer sample

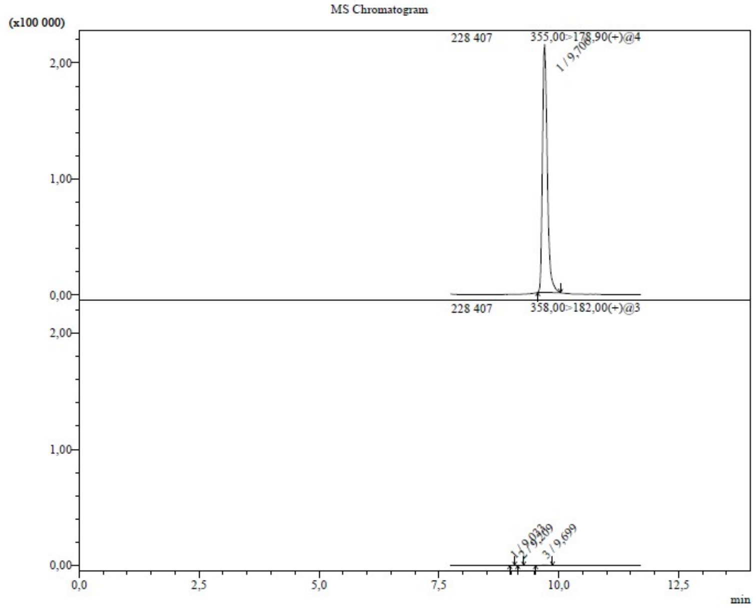

^{1}H and ^{13}C NMR spectra

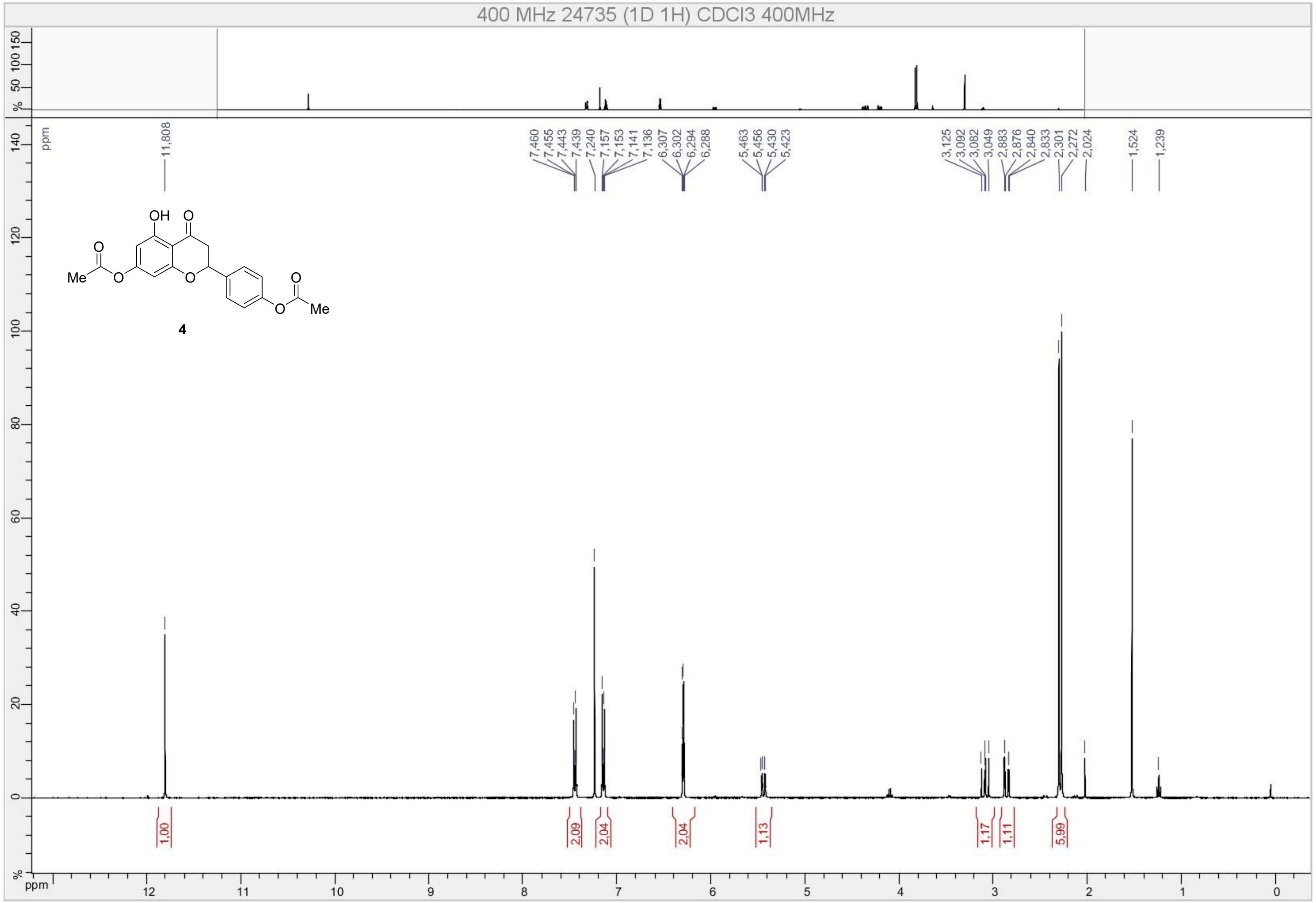
400 MHz 24735 (1D 1H) CDCl3 400MHz
OH
O
Me
O
O
O
O
Me
4
11,808
7,460
7,455
7,443
7,439
7,240
7,157
7,153
7,141
7,136
6,307
6,302
6,294
6,288
5,463
5,456
5,430
5,423
3,125
3,092
3,082
3,049
2,883
2,876
2,840
2,833
2,301
2,272
2,024
1,524
1,239
1,00
2,09
2,04
2,04
1,13
1,17
1,11
5,99
ppm

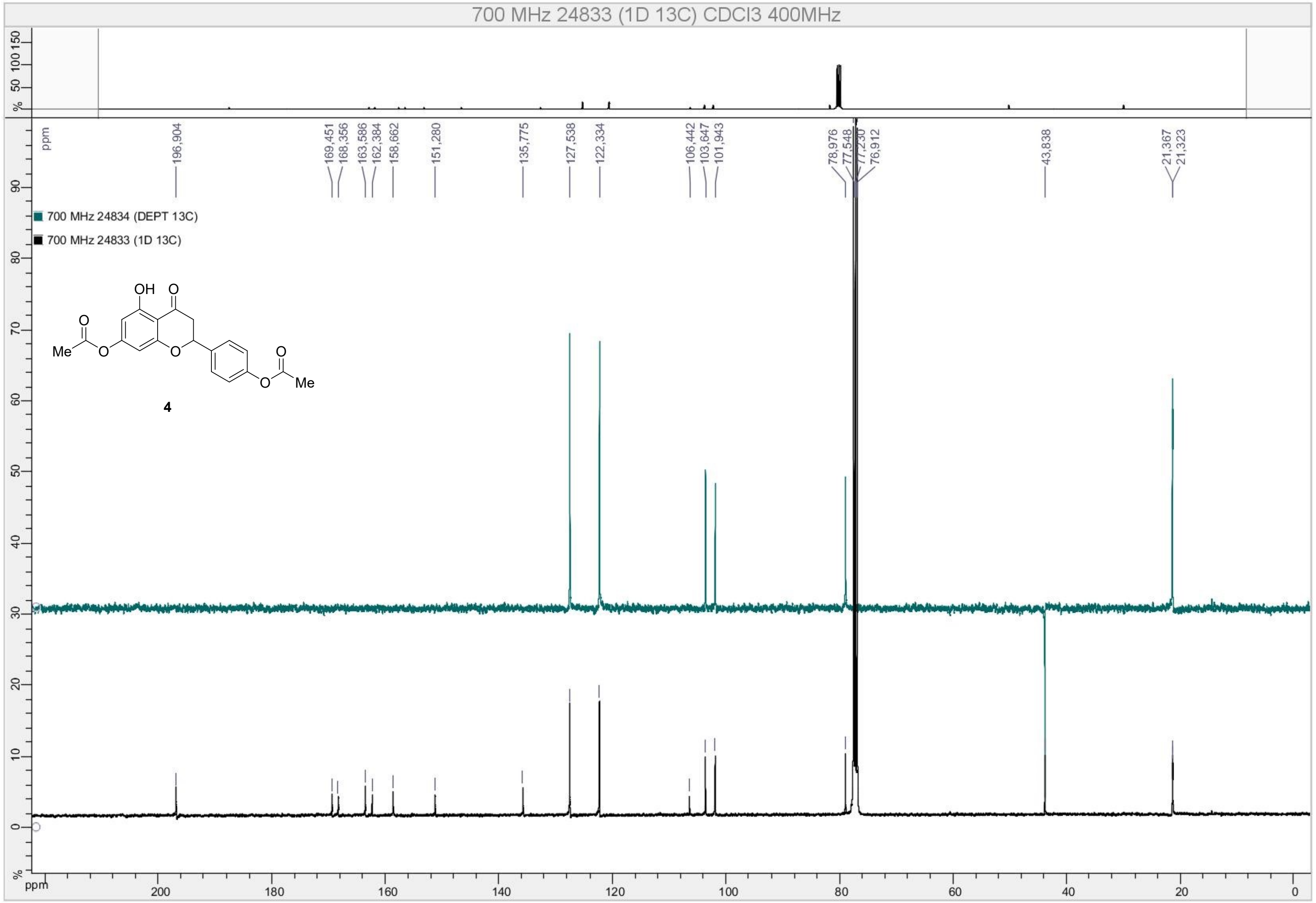

700 MHz 24833 (1D 13C) CDCl3 400MHz
700 MHz 24834 (DEPT 13C)
700 MHz 24833 (1D 13C)
196,904
169,451
168,356
163,586
162,384
158,662
151,280
135,775
127,538
122,334
106,442
103,647
101,943
78,976
77,548
77,230
76,912
43,838
21,367
21,323
OH
O
Me
O
O
O
O
Me
4
ppm
%

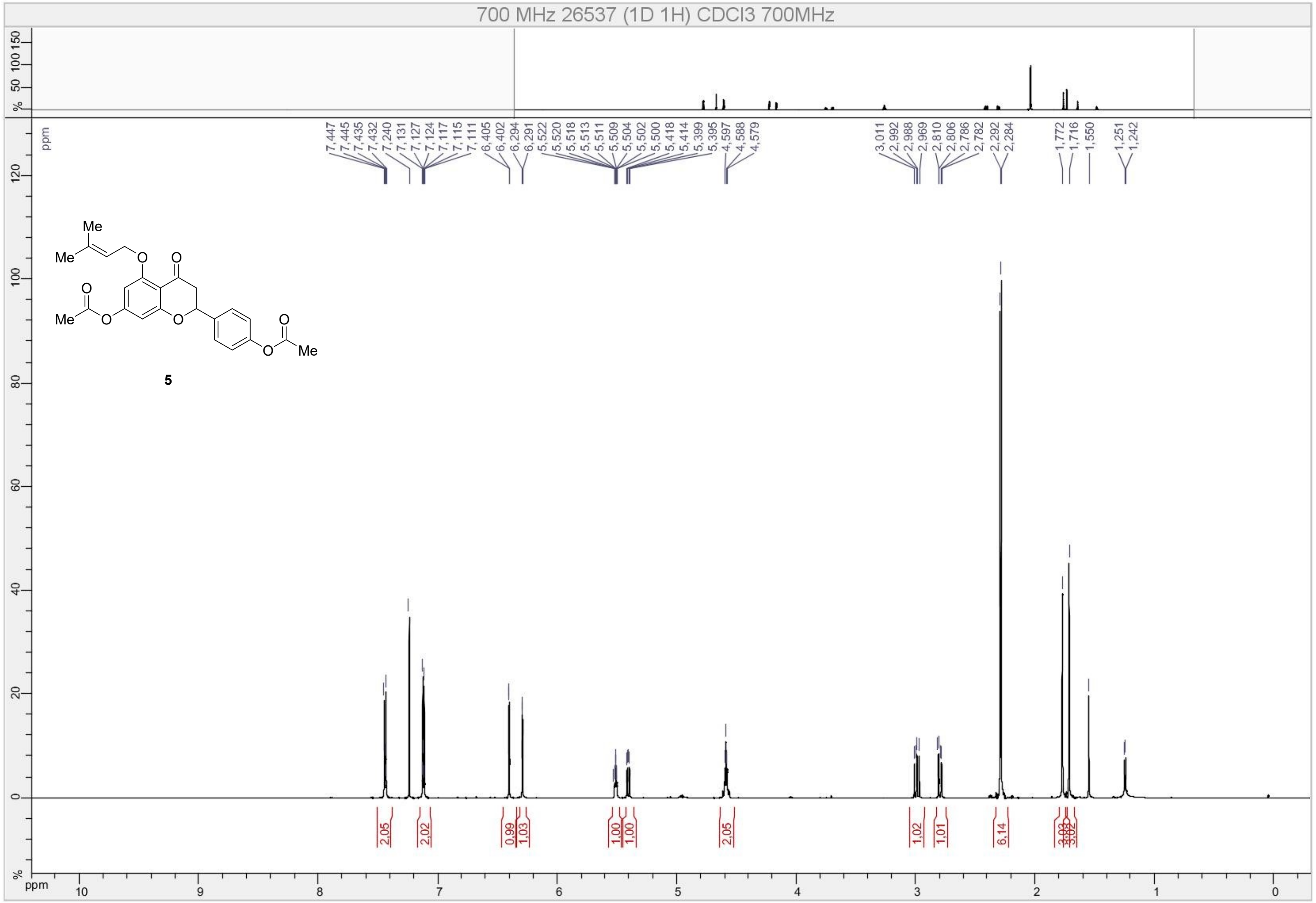

700 MHz 26537 (1D 1H) CDCl3 700MHz
7,447 7,445 7,435 7,432 7,240 7,131 7,127 7,124 7,117 7,115 7,111 6,405 6,402 6,294 6,291 5,522 5,520 5,518 5,513 5,511 5,509 5,504 5,502 5,500 5,418 5,414 5,399 5,395 4,597 4,588 4,579 3,011 2,992 2,988 2,969 2,810 2,806 2,786 2,782 2,292 2,284 1,772 1,716 1,550 1,251 1,242
2,05 2,02 0,99 1,03 1,00 1,00 2,05 1,02 1,01 6,14
Me
Me
O
O
O
O
Me
O
O
O
Me
5
ppm
10 9 8 7 6 5 4 3 2 1 0

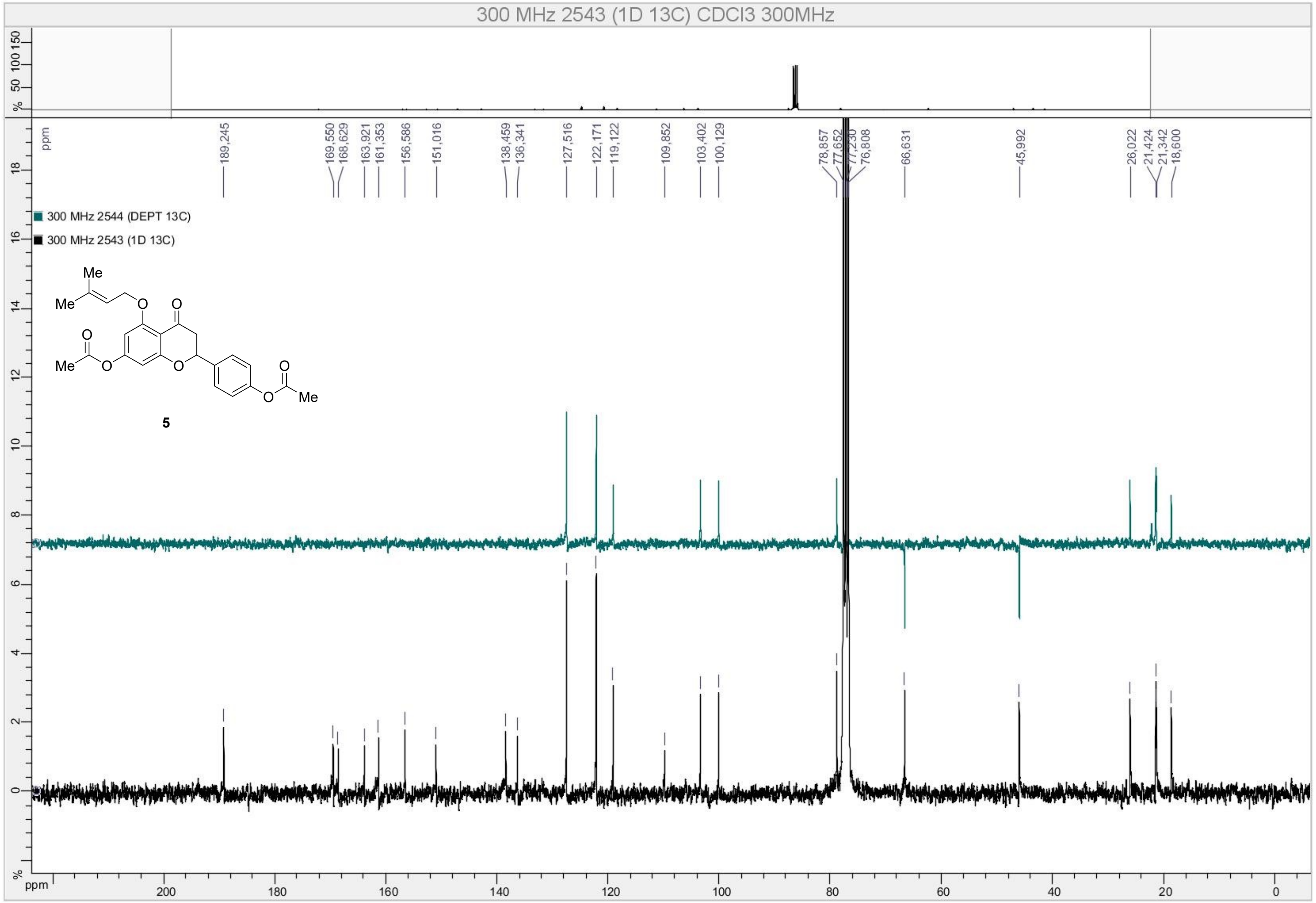
300 MHz 2543 (1D 13C) CDCl3 300MHz
300 MHz 2544 (DEPT 13C)
300 MHz 2543 (1D 13C)
189,245
169,550
168,629
163,921
161,353
156,586
151,016
138,459
136,341
127,516
122,171
119,122
109,852
103,402
100,129
78,857
77,652
77,230
76,808
66,631
45,992
26,022
21,424
21,342
18,600
Me
Me
O
O
O
Me
O
O
O
O
Me
5
ppm

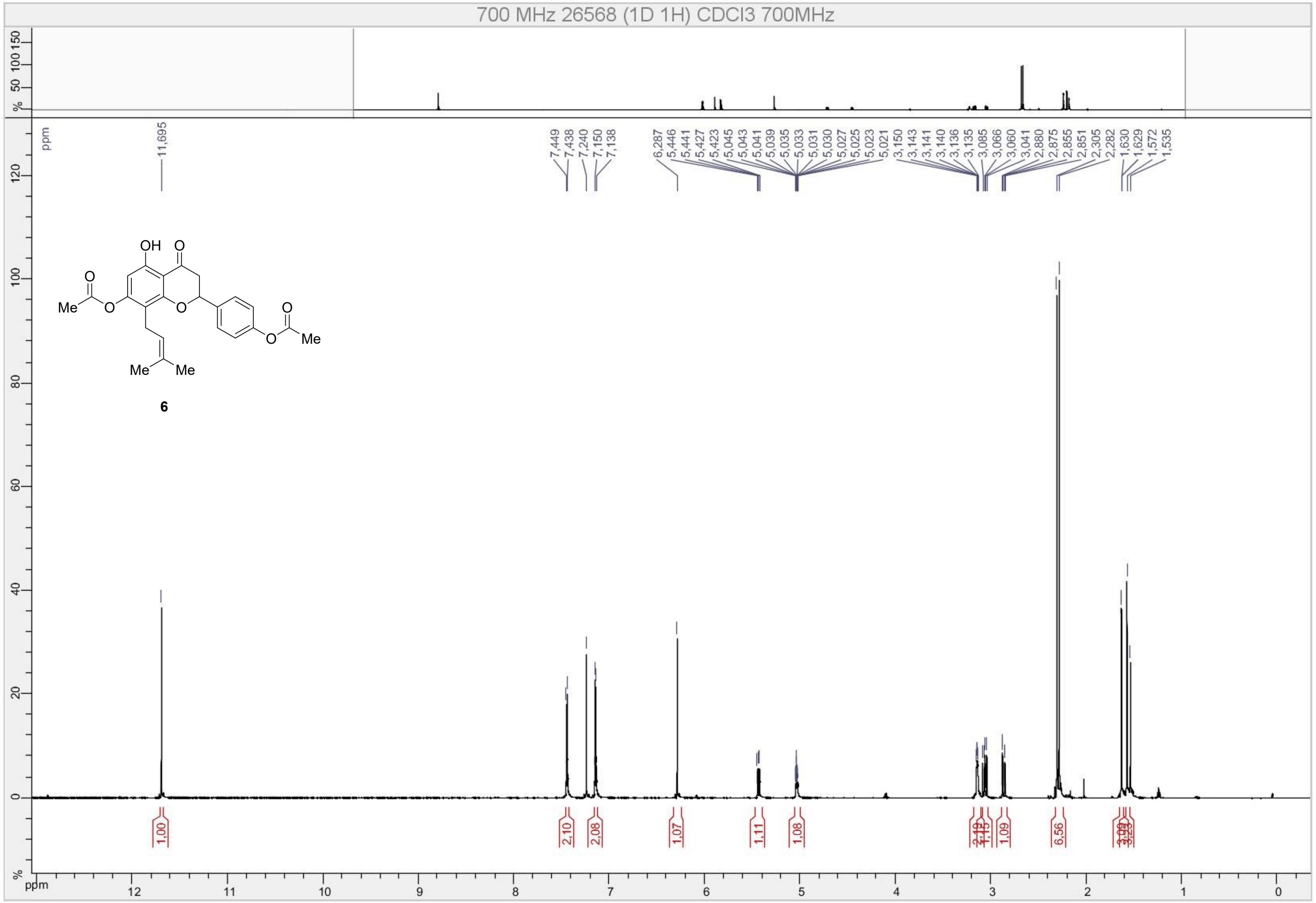
700 MHz 26568 (1D 1H) CDCl3 700MHz
OH
O
O
Me
O
O
O
O
Me
Me
Me
6
11,695
7,449
7,438
7,240
7,150
7,138
6,287
5,446
5,441
5,427
5,423
5,045
5,043
5,041
5,039
5,035
5,033
5,031
5,030
5,027
5,025
5,023
5,021
3,150
3,143
3,141
3,140
3,136
3,135
3,085
3,066
3,060
3,041
2,880
2,875
2,855
2,851
2,305
2,282
1,630
1,629
1,572
1,535
1,00
2,10
2,08
1,07
1,11
1,08
1,09
6,56
ppm

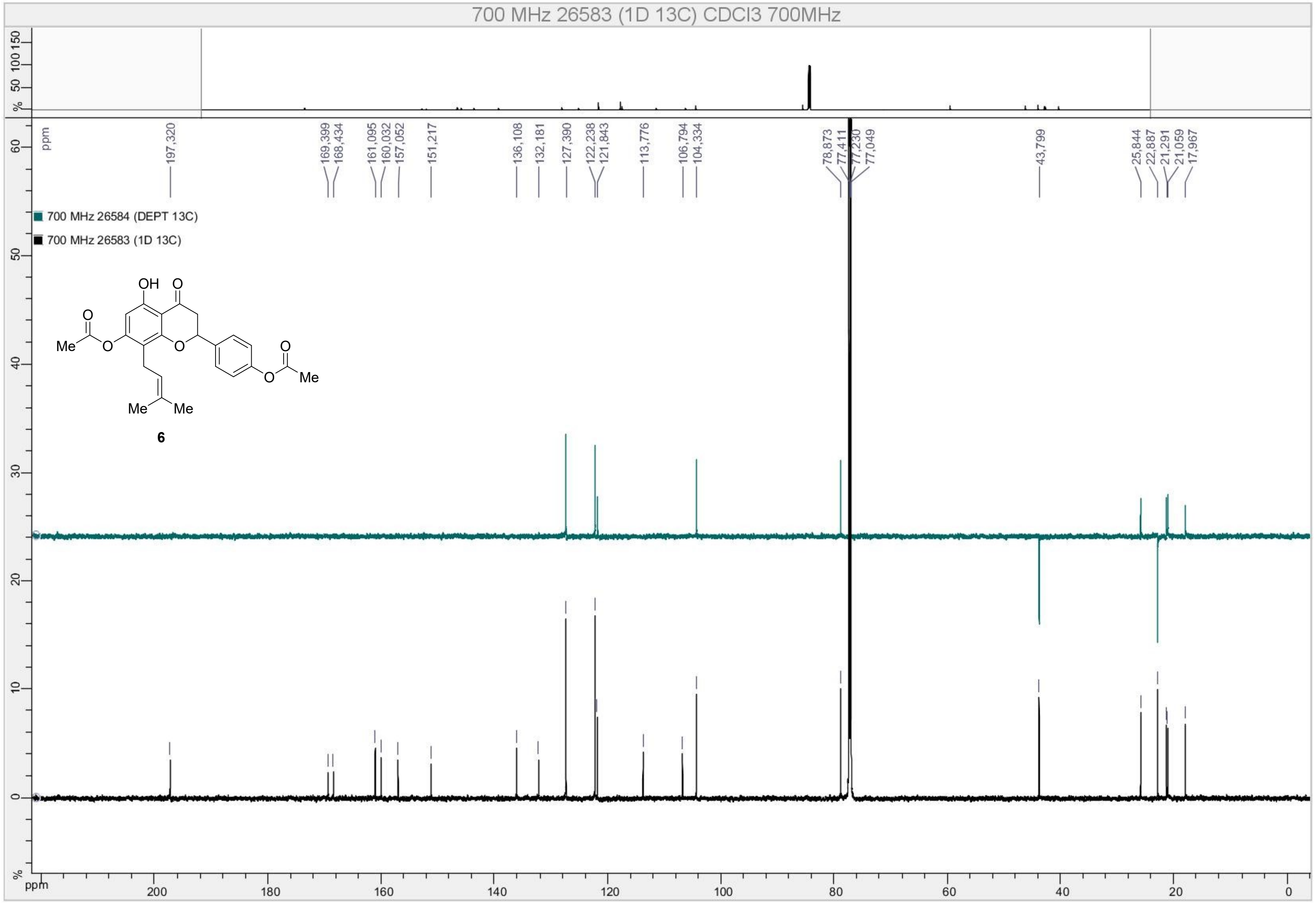
700 MHz 26583 (1D 13C) CDCl3 700MHz
700 MHz 26584 (DEPT 13C)
700 MHz 26583 (1D 13C)
197,320
169,399
168,434
161,095
160,032
157,052
151,217
136,108
132,181
127,390
122,238
121,843
113,776
106,794
104,334
78,873
77,411
77,230
77,049
43,799
25,844
22,887
21,291
21,059
17,967
OH
O
Me
O
O
O
O
O
Me
Me
Me
6
ppm
%

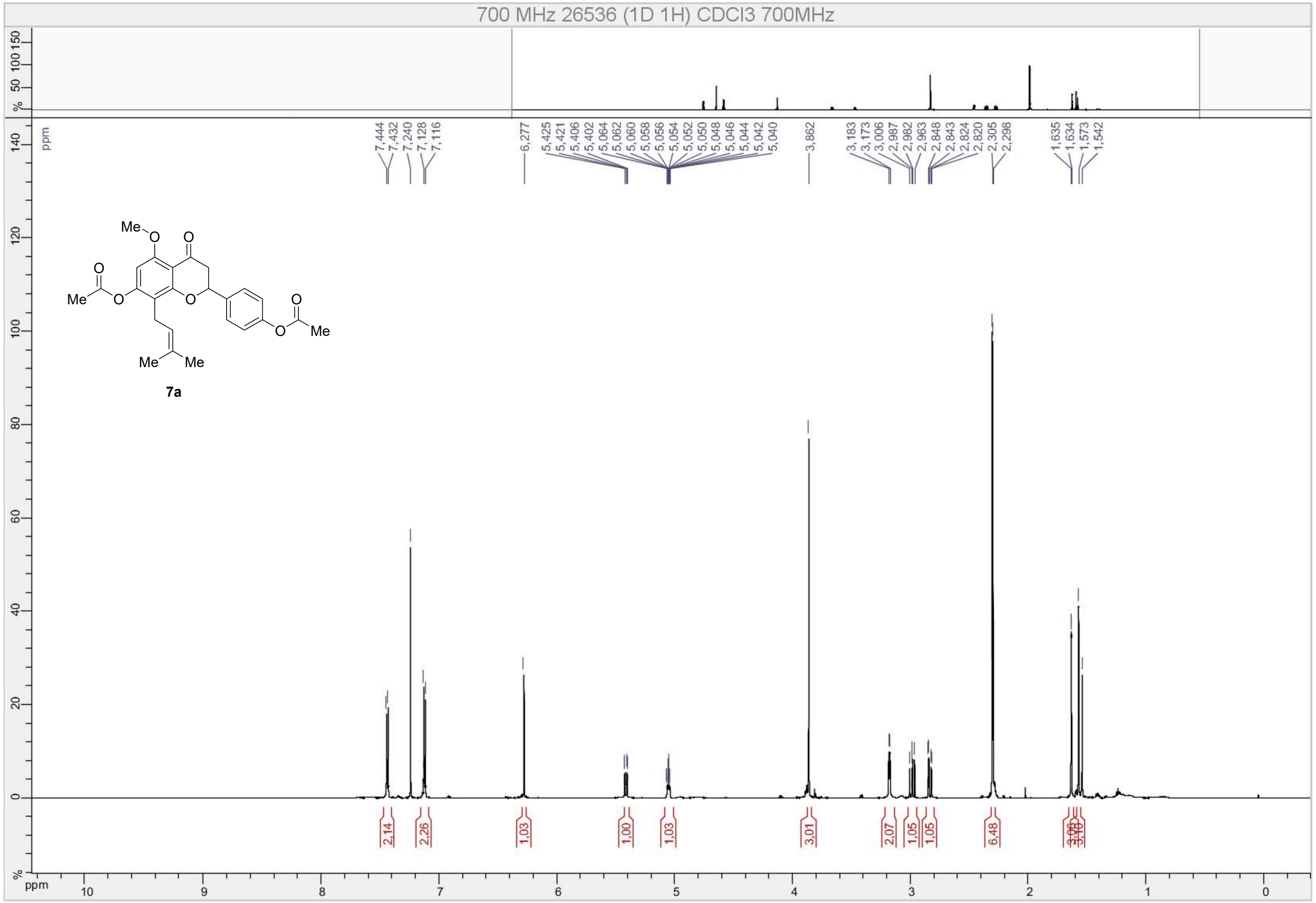
700 MHz 26536 (1D 1H) CDCl3 700MHz
7a
Me
O
O
O
Me
O
O
Me
Me
O
O
Me
7,444
7,432
7,240
7,128
7,116
6,277
5,425
5,421
5,406
5,402
5,064
5,062
5,060
5,058
5,056
5,054
5,052
5,050
5,048
5,046
5,044
5,042
5,040
3,862
3,183
3,173
3,006
2,987
2,982
2,963
2,848
2,843
2,824
2,820
2,305
2,298
1,635
1,634
1,573
1,542
2,14
2,26
1,03
1,00
1,03
3,01
2,07
1,05
1,05
6,48
ppm

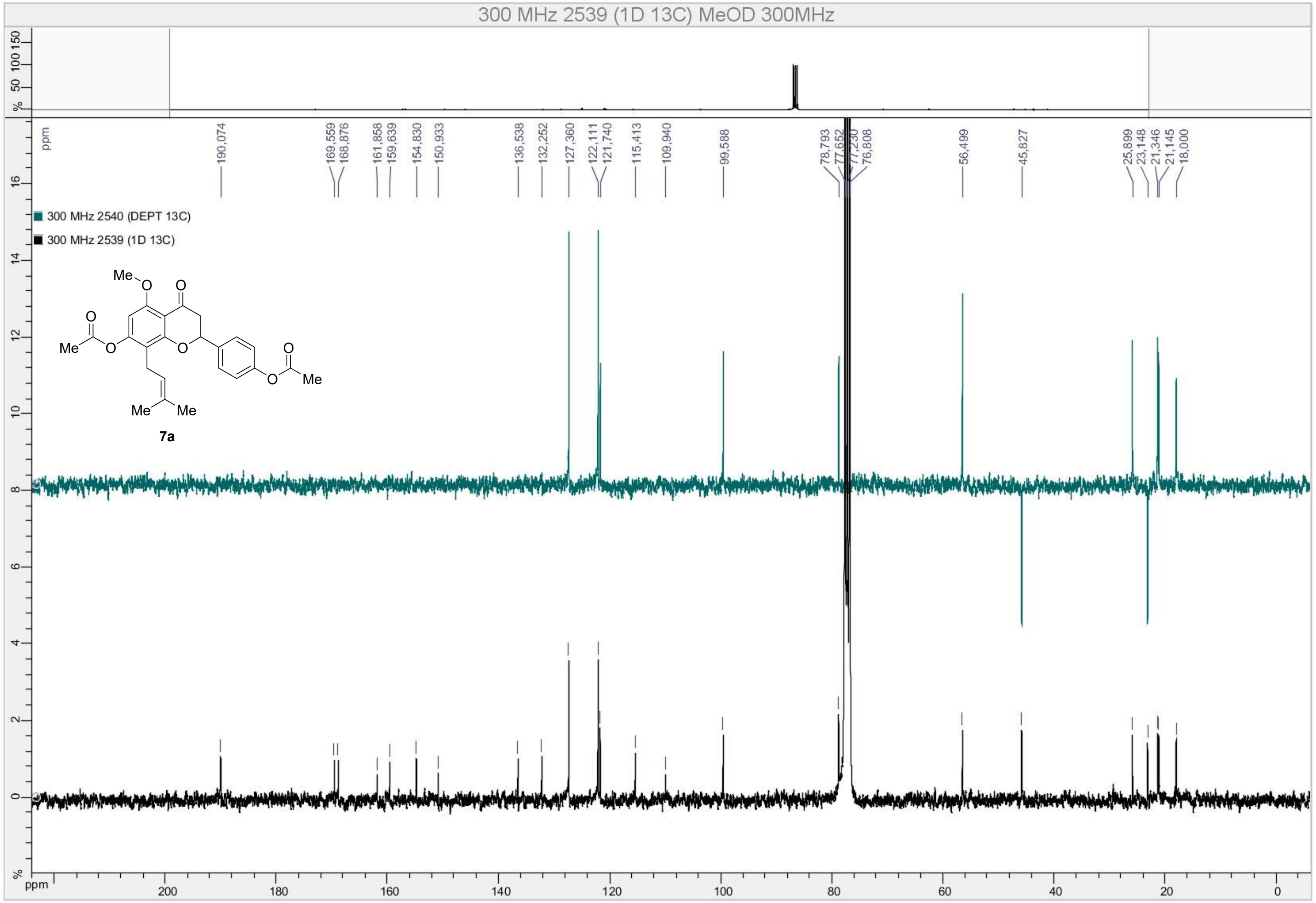
300 MHz 2539 (1D 13C) MeOD 300MHz
300 MHz 2540 (DEPT 13C)
300 MHz 2539 (1D 13C)
190,074
169,559
168,876
161,858
159,639
154,830
150,933
136,538
132,252
127,360
122,111
121,740
115,413
109,940
99,588
78,793
77,652
77,230
76,808
56,499
45,827
25,899
23,148
21,346
21,145
18,000
Me
O
O
O
Me
O
O
O
O
Me
Me
Me
O
Me
7a
ppm

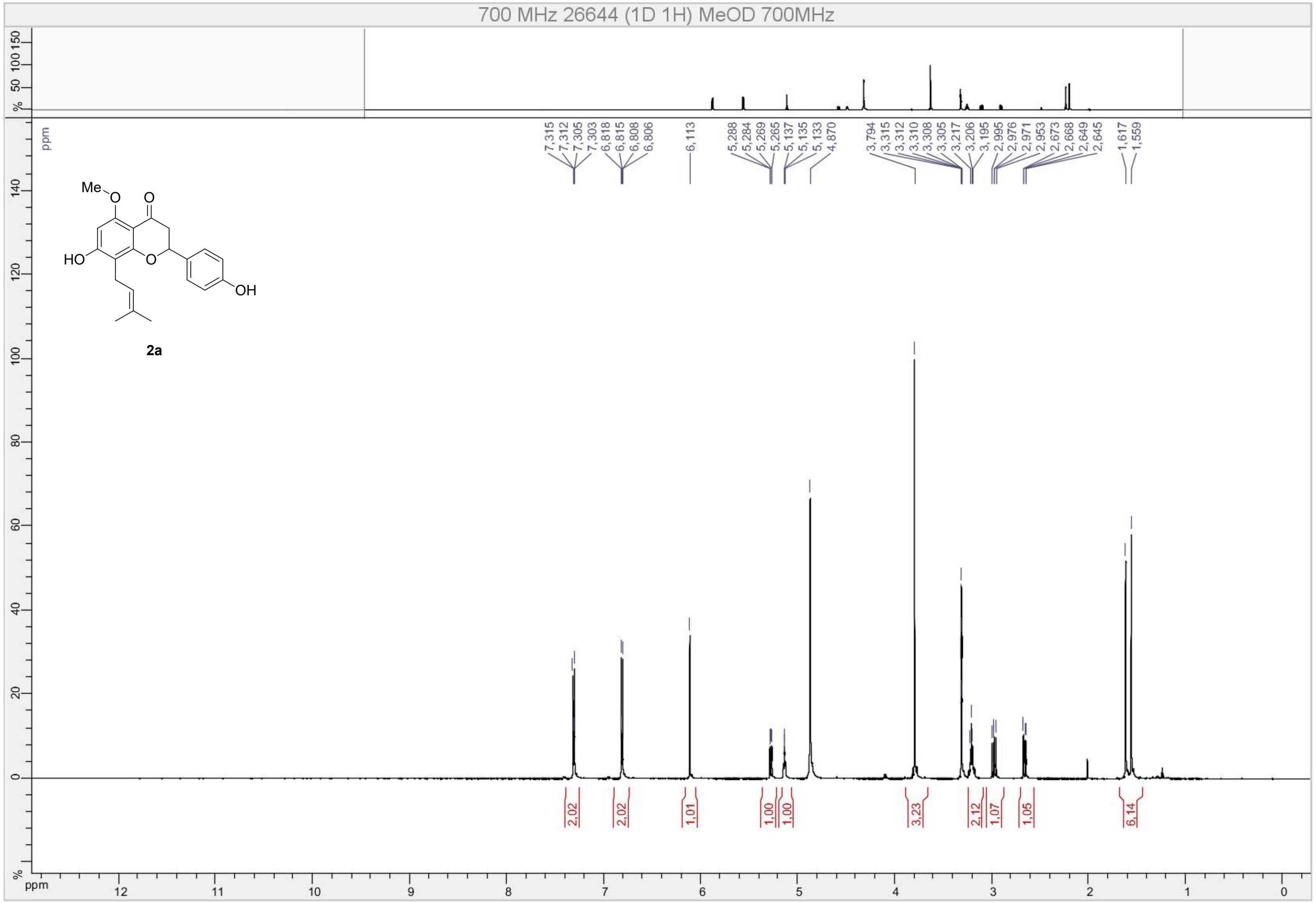
700 MHz 26644 (1D 1H) MeOD 700MHz
7,315
7,312
7,305
7,303
6,818
6,815
6,808
6,806
6,113
5,288
5,284
5,269
5,265
5,137
5,135
5,133
4,870
3,794
3,315
3,312
3,310
3,308
3,305
3,217
3,206
3,195
2,995
2,976
2,971
2,953
2,673
2,668
2,649
2,645
1,617
1,559
2,02
2,02
1,01
1,00
1,00
3,23
2,12
1,07
1,05
6,14
Me
O
O
HO
O
OH
2a
ppm
%

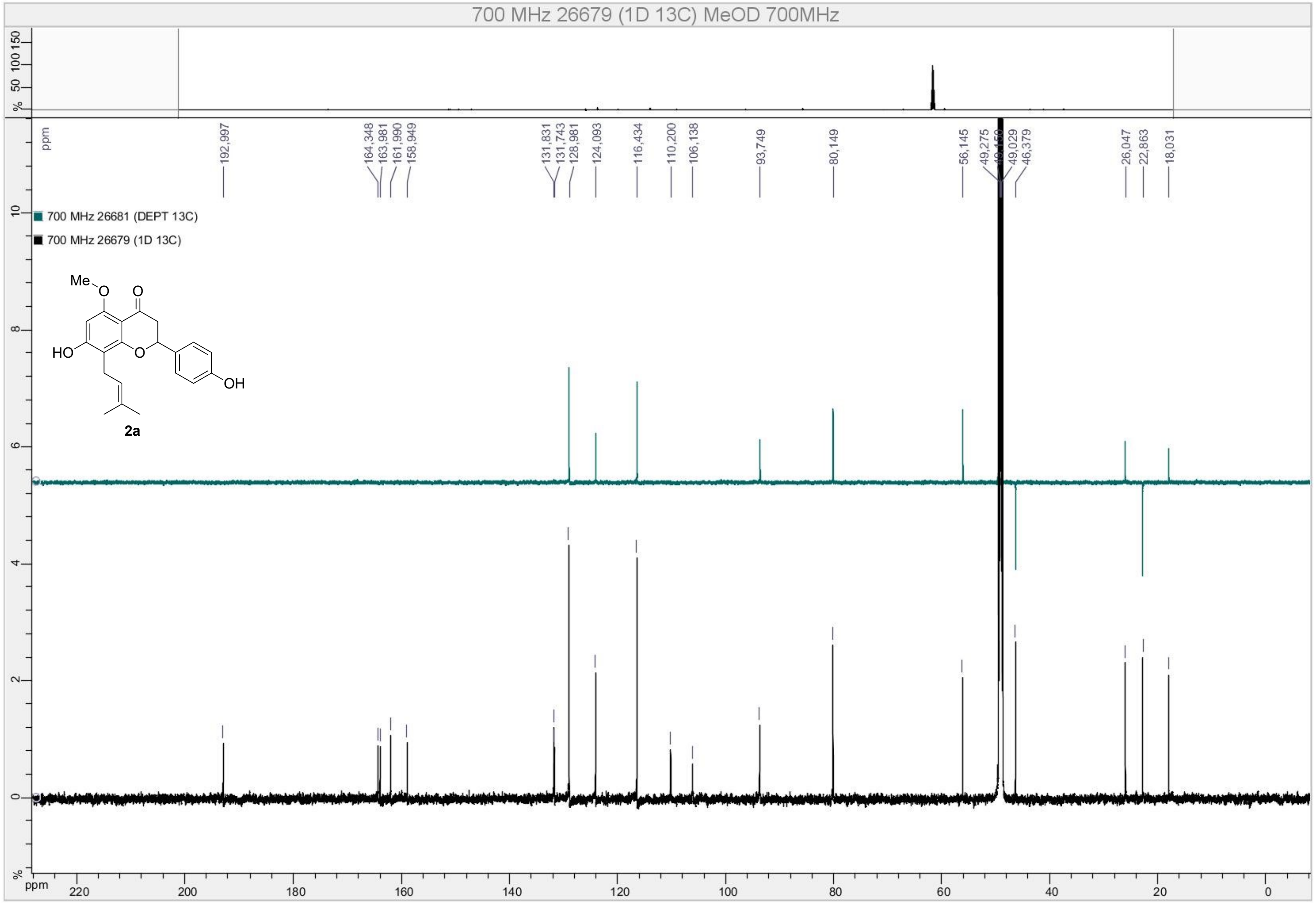
700 MHz 26679 (1D 13C) MeOD 700MHz
700 MHz 26681 (DEPT 13C)
700 MHz 26679 (1D 13C)
192,997
164,348
163,981
161,990
158,949
131,831
131,743
128,981
124,093
116,434
110,200
106,138
93,749
80,149
56,145
49,275
49,029
46,379
26,047
22,863
18,031
Me
O
O
HO
O
OH
2a
ppm
%

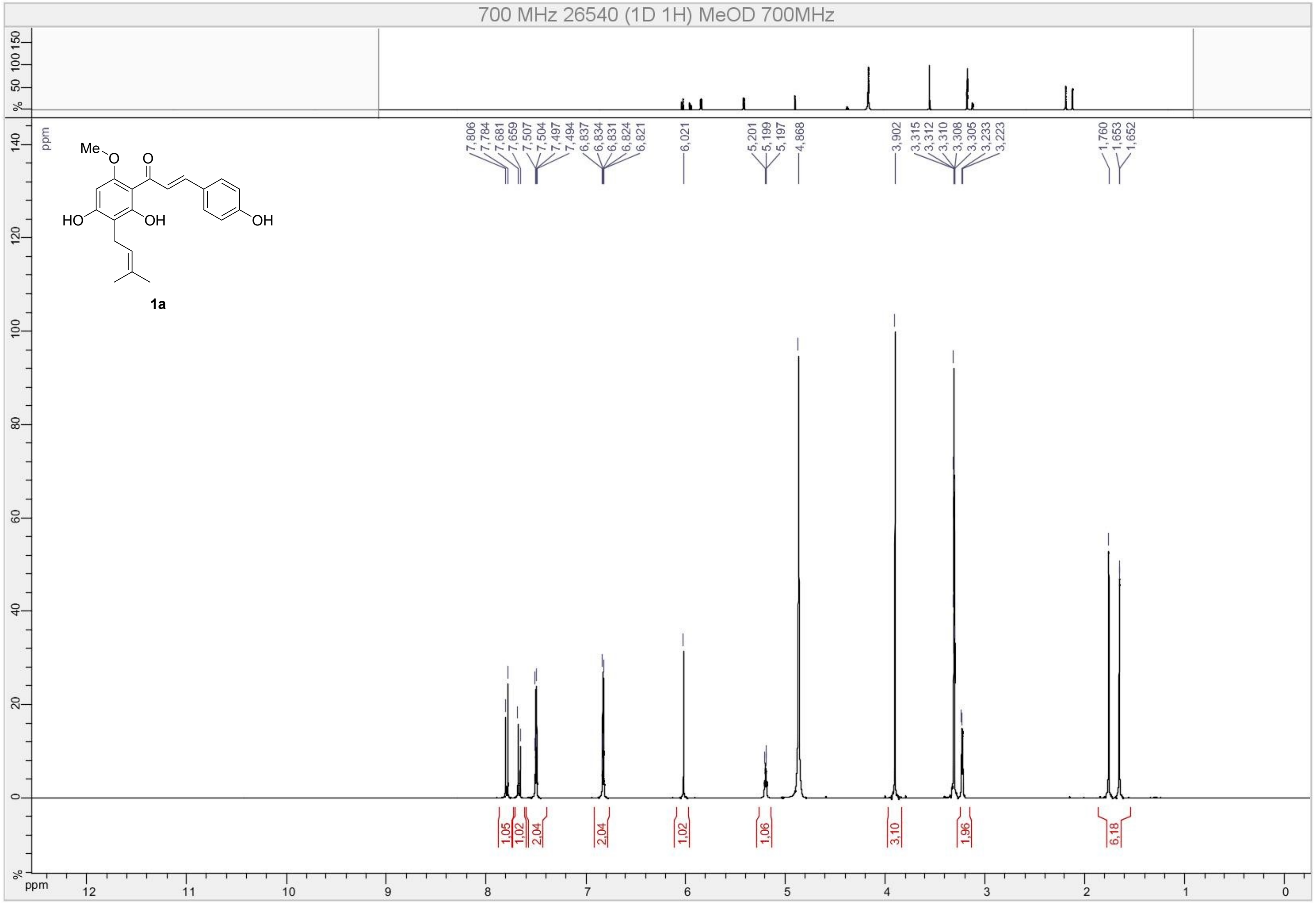
700 MHz 26540 (1D 1H) MeOD 700MHz
7,806
7,784
7,681
7,659
7,507
7,504
7,497
7,494
6,837
6,834
6,831
6,824
6,821
6,021
5,201
5,199
5,197
4,868
3,902
3,315
3,312
3,310
3,308
3,305
3,233
3,223
1,760
1,653
1,652
1,05
1,02
2,04
2,04
1,02
1,06
3,10
1,96
6,18
Me
O
O
HO
OH
OH
1a
ppm
%

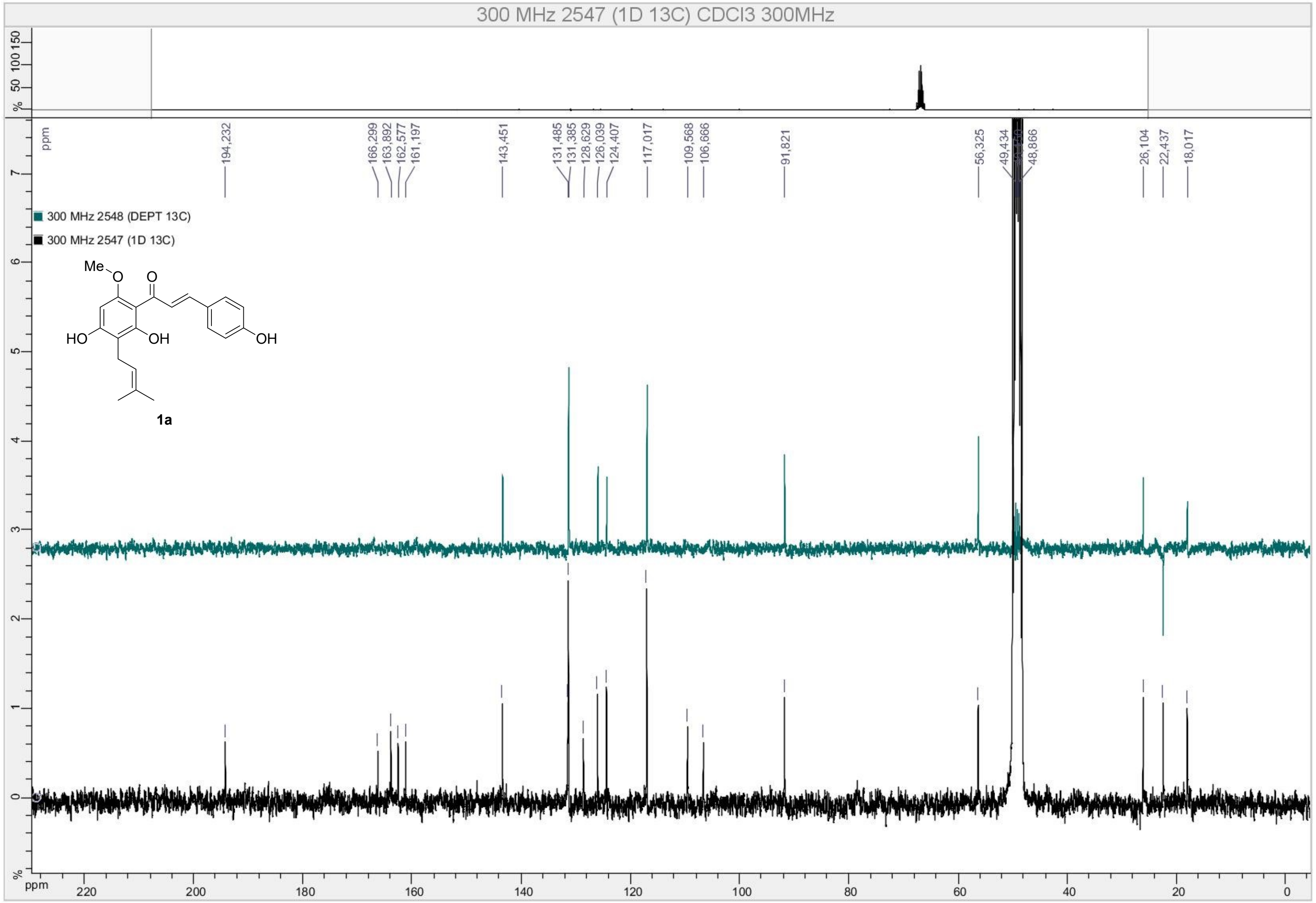
300 MHz 2547 (1D 13C) CDCl3 300MHz
300 MHz 2548 (DEPT 13C)
300 MHz 2547 (1D 13C)
194,232
166,299
163,892
162,577
161,197
143,451
131,485
131,385
128,629
126,039
124,407
117,017
109,568
106,666
91,821
56,325
49,434
48,866
26,104
22,437
18,017
Me
O
O
HO
OH
OH
1a
ppm
%

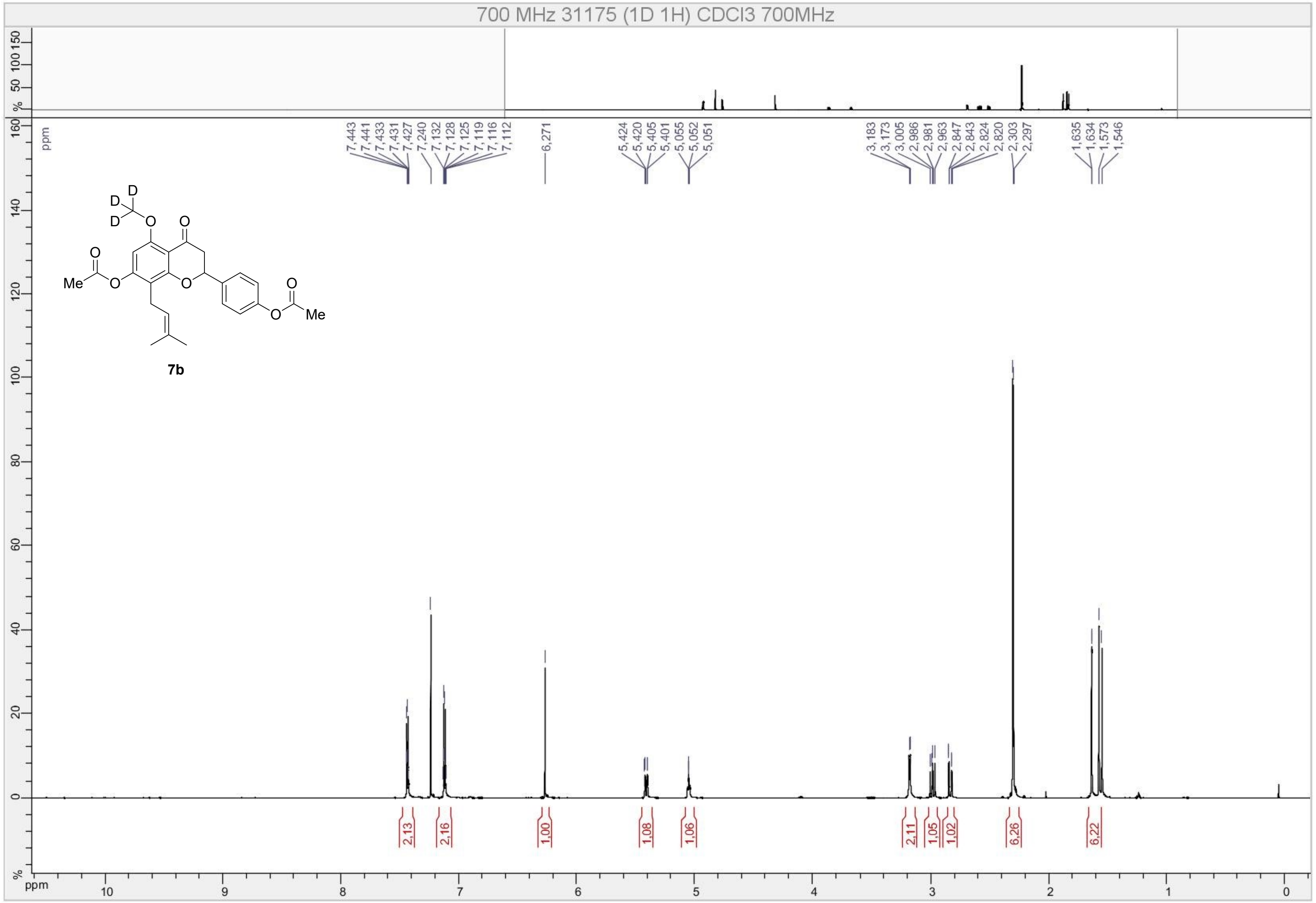
700 MHz 31175 (1D 1H) CDCl3 700MHz
7,443 7,441 7,433 7,431 7,427 7,240 7,132 7,128 7,125 7,119 7,116 7,112
6,271
5,424 5,420 5,405 5,401 5,055 5,052 5,051
3,183 3,173 3,005 2,986 2,981 2,963 2,847 2,843 2,824 2,820 2,303 2,297
1,635 1,634 1,573 1,546
2,13 2,16 1,00 1,08 1,06 2,11 1,05 1,02 6,26 6,22
D
D
D
O
O
O
Me
O
O
O
O
Me
7b
ppm
%

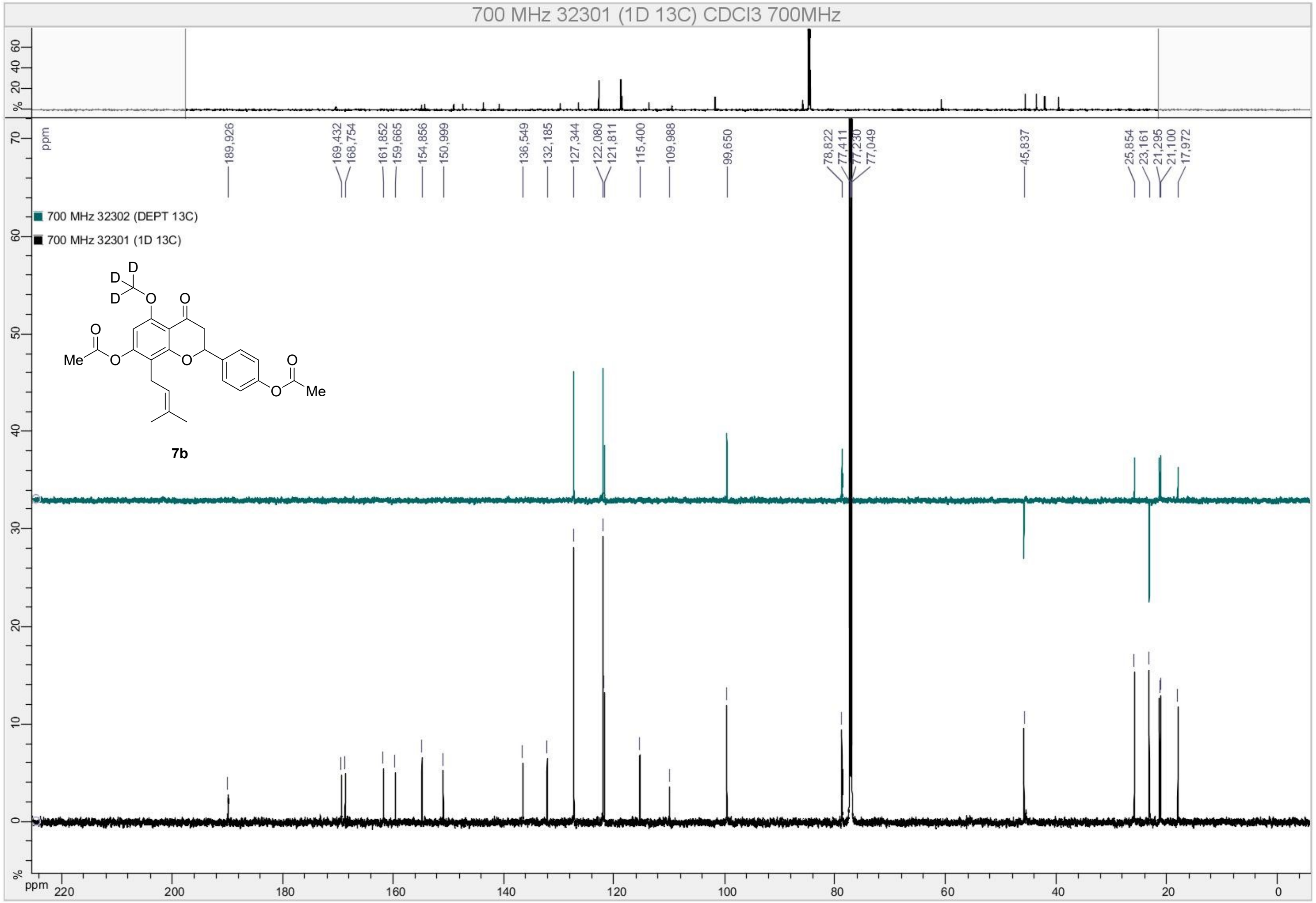
700 MHz 32301 (1D 13C) CDCl3 700MHz
700 MHz 32302 (DEPT 13C)
700 MHz 32301 (1D 13C)
189,926
169,432
168,754
161,852
159,665
154,856
150,999
136,549
132,185
127,344
122,080
121,811
115,400
109,988
99,650
78,822
77,411
77,230
77,049
45,837
25,854
23,161
21,295
21,100
17,972
7b
ppm
%

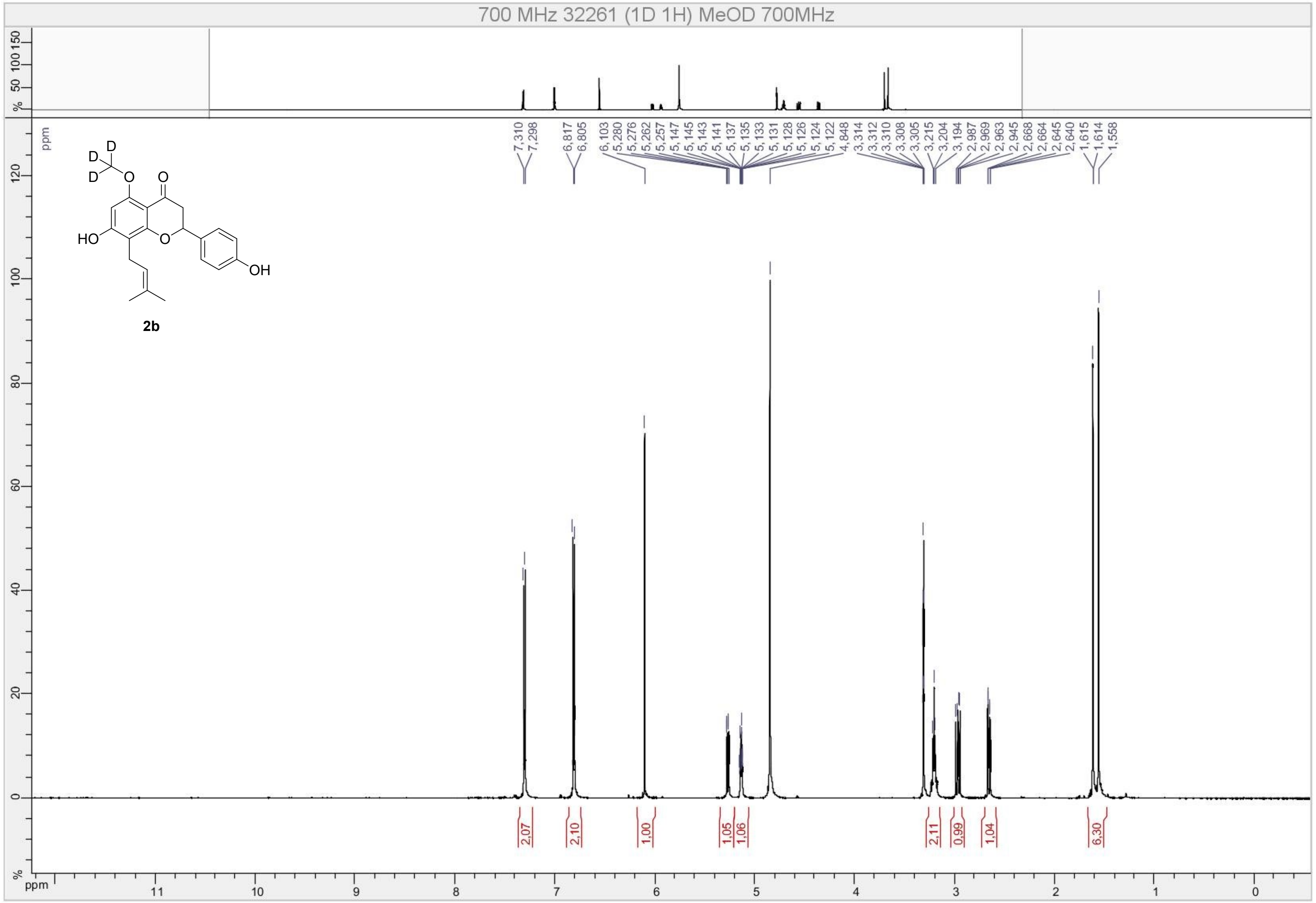

700 MHz 32261 (1D 1H) MeOD 700MHz
2b
7,310
7,298
6,817
6,805
6,103
5,280
5,276
5,262
5,257
5,147
5,145
5,143
5,141
5,137
5,135
5,135
5,133
5,131
5,128
5,126
5,124
5,122
4,848
3,314
3,312
3,310
3,308
3,305
3,215
3,204
3,194
2,987
2,969
2,963
2,945
2,668
2,664
2,645
2,640
1,615
1,614
1,558
2,07
2,10
1,00
1,05
1,06
2,11
0,99
1,04
6,30
ppm

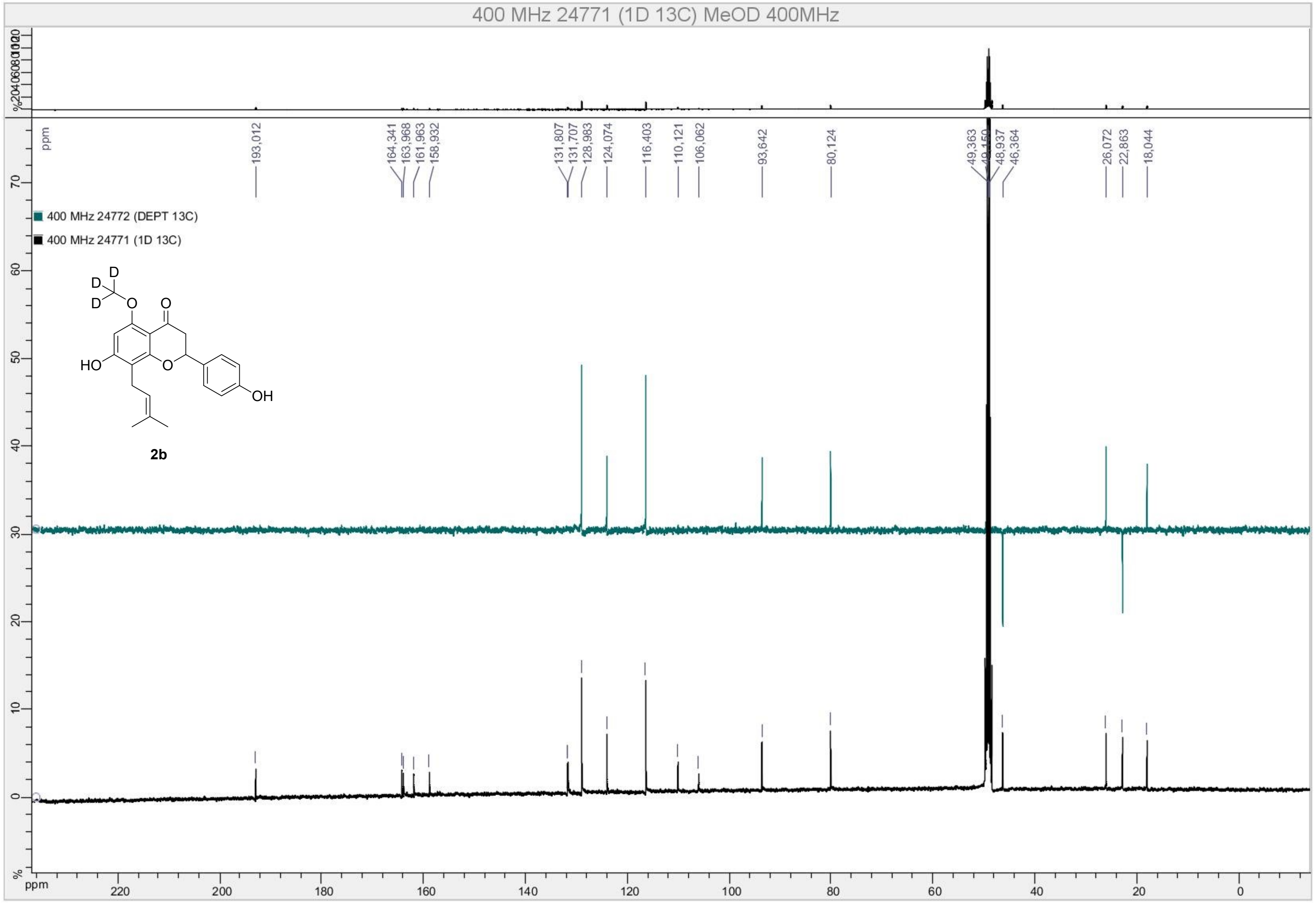

400 MHz 24771 (1D 13C) MeOD 400MHz
193,012
164,341
163,968
161,963
158,932
131,807
131,707
128,983
124,074
116,403
110,121
106,062
93,642
80,124
49,363
48,937
46,364
26,072
22,863
18,044
400 MHz 24772 (DEPT 13C)
400 MHz 24771 (1D 13C)
2b
ppm

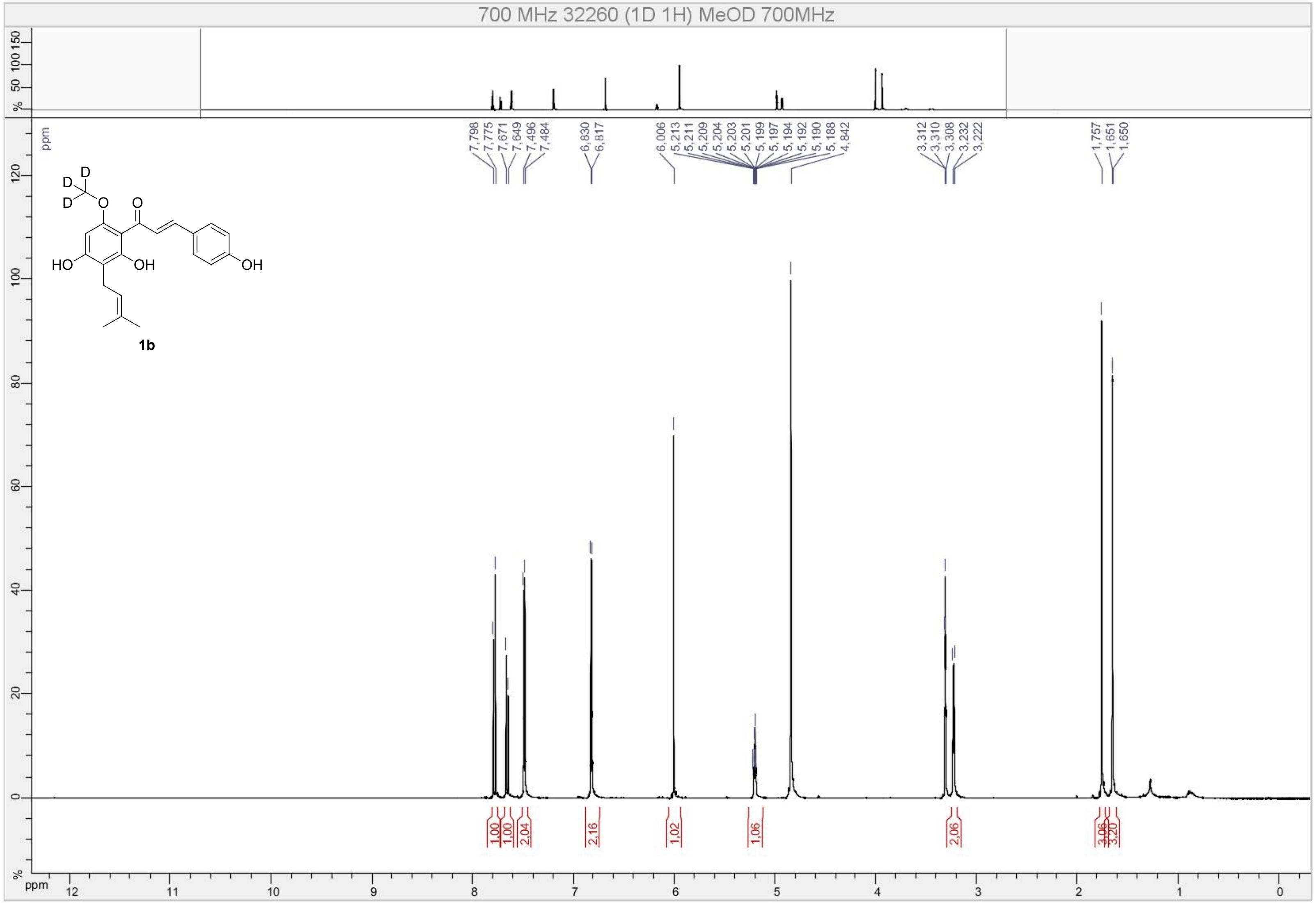
700 MHz 32260 (1D 1H) MeOD 700MHz
7,798
7,775
7,671
7,649
7,496
7,484
6,830
6,817
6,006
5,213
5,211
5,209
5,204
5,203
5,201
5,199
5,197
5,194
5,192
5,190
5,188
4,842
3,312
3,310
3,308
3,232
3,222
1,757
1,651
1,650
1,00
1,00
2,04
2,16
1,02
1,06
2,06
3,06
3,20
HO
OH
OH
O
D
D
D
1b
ppm
%

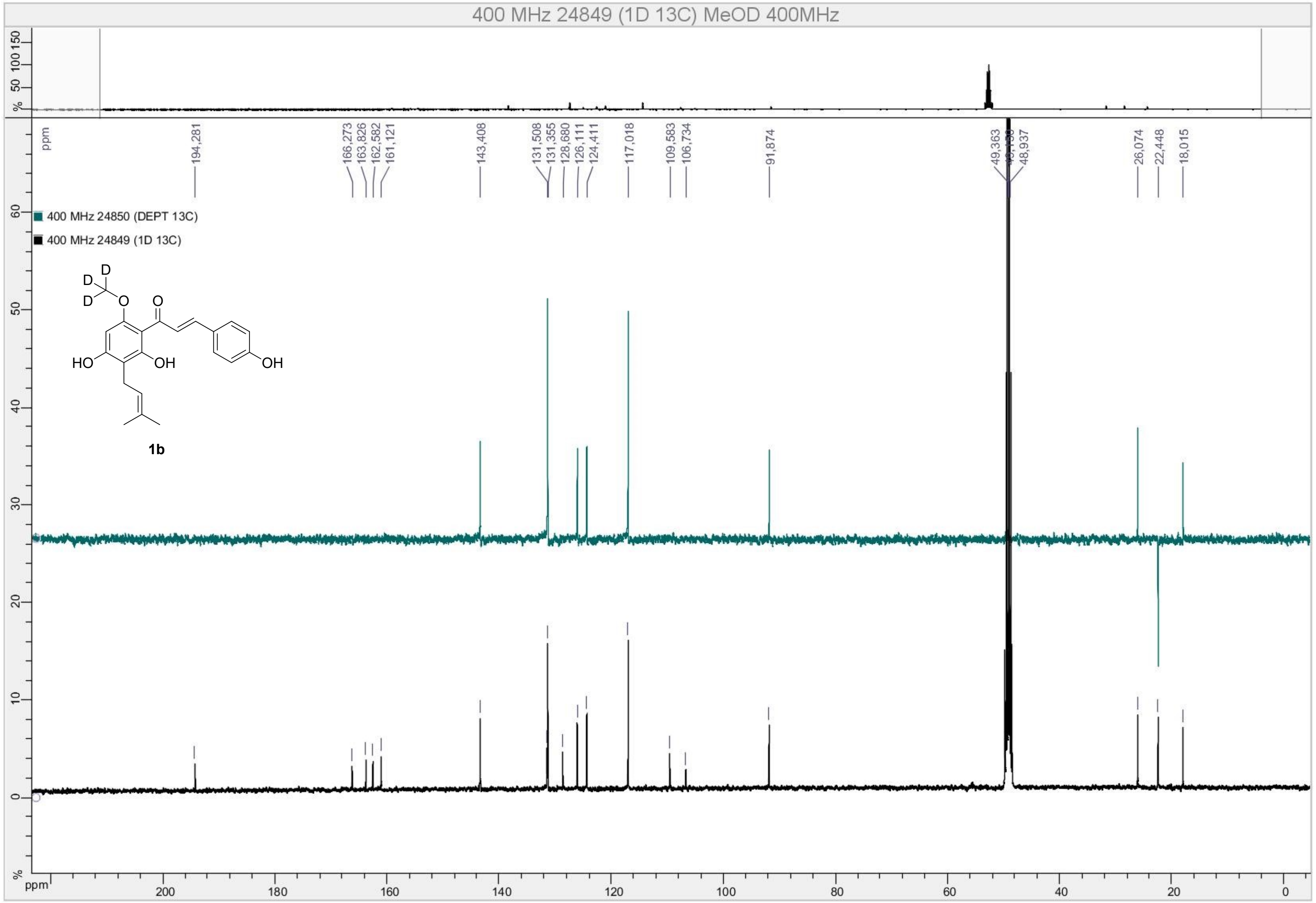
400 MHz 24849 (1D 13C) MeOD 400MHz
194,281
166,273
163,826
162,582
161,121
143,408
131,508
131,355
128,680
126,111
124,411
117,018
109,583
106,734
91,874
49,363
48,937
26,074
22,448
18,015
400 MHz 24850 (DEPT 13C)
400 MHz 24849 (1D 13C)
1b
ppm
